# Skeletal muscle dysfunction induced by cancer immunotherapy: mechanistics insights and surgical implications

**DOI:** 10.3389/fimmu.2026.1787374

**Published:** 2026-03-31

**Authors:** Shuang Ma, Yu Sun, Shang Sui, Xinwei Wu, Xiaolin Wu, Huixian Li

**Affiliations:** 1 School of Information Science and Engineering, Shenyang Ligong University, Shenyang, China; 2 St. John’s Killmarnock School, Waterloo Region (Breslau), Ontario, ON, Canada; 3 School of Mathematics and Statistics, Liaoning University, Shenyang, China; 4 Department of Anesthesiology, National Cancer Center/National Clinical Research Center for Cancer/Cancer Hospital, Chinese Academy of Medical Sciences and Peking Union Medical College, Beijing, China

**Keywords:** cytokines, immunotherapy, metabolic dysfunction, skeletal muscle injury, tumor microenvironment

## Abstract

Cancer immunotherapy has revolutionized cancer treatment, yet its effects extend far beyond tumor eradication. Accumulating evidence indicates that immunotherapy-associated skeletal muscle dysfunction represents a complex, cross-organ pathological process driven by dynamic crosstalk between the tumor microenvironment and peripheral tissues. Rather than an isolated adverse event, muscle injury emerges from integrated mechanisms including inflammatory cytokine spillover, aberrant immune cell infiltration, metabolic reprogramming, vascular dysfunction, and impaired regenerative signaling. Central to this process is the sustained activation of the NF-κB and JAK/STAT3 axes, which links systemic immune activation to ubiquitin-proteasome-mediated proteolysis, mitochondrial dysfunction, and suppression of anabolic pathways. Meanwhile, metabolic coupling between tumor glycolysis and skeletal muscle energetics establishes a bidirectional feedback loop that exacerbates catabolism and compromises antitumor immunity. Clinically, baseline sarcopenia and therapy-induced myotoxicity reciprocally impair immunotherapeutic efficacy, forming a self-reinforcing cycle that limits treatment continuity and long-term survival. Advances in multimodal imaging, including PET/CT, shear wave elastography, and dynamic contrast-enhanced MRI, combined with artificial intelligence-driven quantitative analysis, provide a noninvasive framework to decode metabolic, mechanical, and vascular signatures of muscle vulnerability. Emerging interventions targeting inflammatory signaling, metabolic imbalance, vascular dysregulation, and regenerative pathways offer promising strategies to dissociate antitumor efficacy from systemic toxicity. Future research should prioritize longitudinal, multi-omics-integrated, and imaging-guided approaches to clarify causal hierarchies and enable precision risk stratification. Bridging mechanistic insight with perioperative and translational strategies will be essential to achieving comprehensive cancer care in the immunotherapy era.

## 1 Introduction

The tumor microenvironment (TME) is a complex and dynamic system composed of tumor, immune, and stromal cells, as well as the extracellular matrix (1). It is essential for the initiation, progression, and therapeutic response of tumors (2). Immunotherapy is an emerging cancer treatment option that augments or stimulates the host immune system to identify and eradicate tumor cells, thereby demonstrating substantial clinical potential (1). The interaction between TME and immunotherapy is complex, affecting tumor cell behavior and significantly affecting skeletal muscle tissue (3). Early cancer research primarily focused on tumor cells. Scientists have increasingly acknowledged that the surrounding microenvironment is as essential as tumor progression (4). This transition from a tumor cell-centric view to one that includes immune and stromal cells and the extracellular matrix has enhanced our understanding of TME (5). Initially, tumors were believed to result from uncontrolled cell proliferation. However, the discovery of immune and stromal components within tumors has highlighted the complexity of the TME (6). Cancer-associated fibroblasts (CAFs) facilitate tumor cell proliferation and invasion by secreting extracellular matrix (ECM) components and cytokines, including transforming growth factor-β (TGF-β) and platelet-derived growth factor (PDGF) (7). Immune cells within the TME play dual roles. Tumor-infiltrating lymphocytes (TILs; CD8^+^ T cells and natural killer (NK) cells) can identify and eradicate tumor cells, whereas myeloid-derived suppressor cells (MDSCs) and tumor-associated macrophages (TAMs) may facilitate immune evasion through secreting immunosuppressive cytokines (8).

Immunotherapy has emerged as a promising advancement for cancer treatment. Several decades ago, scientists commenced investigations into the potential of utilizing the immune system to combat cancer. However, our understanding of the mechanisms and applications of immunotherapy is limited (9). Recently, significant breakthroughs have been achieved with a deeper understanding of immune-tumor interactions. Immune checkpoint inhibitors, adoptive cell therapy (ACT), and cytokine-based therapies constitute the three major strategies that augment anti-tumor immunity by inhibiting pathways, including Programmed Cell Death Protein 1 (PD-1)/programmed death ligand 1 (PD-L1) and Cytotoxic T-Lymphocyte-Associated Protein 4 (10). These approaches have demonstrated significant potential in clinical settings and have markedly enhanced the prognosis of cancer patients (11). However, activating anti-tumor immune responses through immunotherapy may induce an excessive release of inflammatory cytokines and abnormal immune cell infiltration, culminating in skeletal muscle damage (12). This damage does not occur in isolation but has a close pathological association with cancer cachexia. Cancer cachexia is a systemic metabolic-inflammatory syndrome characterized by progressive skeletal muscle loss, serving as a bridge between TME imbalance and poor response to immunotherapy (13). For patients with pre-existing cachexia, the persistent state of chronic systemic inflammation inhibits the proliferative capacity and cytotoxic function of CD8^+^ T cells and NK cells. Meanwhile, metabolic reprogramming and nutrient substrate exhaustion occurring in muscle tissue under cachectic conditions further impair the metabolic adaptability and effector function of immune cells, thereby contributing to a more immunosuppressive TME and reducing sensitivity to immunotherapy (14). On the other hand, in the process of reshaping the TME and activating anti-tumor immune responses, immunotherapy is often accompanied by elevated cytokine levels and abnormal infiltration of immune cells into peripheral tissues. This systemic inflammatory amplification effect can overactivate catabolic pathways in skeletal muscle, inhibit myogenic differentiation and regeneration, accelerate the net loss of muscle proteins, and further promote the progression of cachexia (15). Thus, a mutually reinforcing pathological cycle is formed, wherein cachexia impairs the efficacy of immunotherapy, while immunotherapy-related inflammation accelerates muscle wasting. This cycle not only significantly affects patients’ physical status and quality of life but also becomes an important clinical obstacle that limits treatment outcomes and long-term survival. This review summarizes recent literature, beginning with an overview of the composition and characteristics of the TME (16). We subsequently examined how immunotherapy alters TME, discussed TME in immunotherapeutic efficacy, and clarified the mechanisms by which immunotherapy induces skeletal muscle injury (15). To enhance the cutting-edge nature and evidence quality of this review, this article mainly focuses on high-quality original studies and authoritative reviews published in the past three years, and systematically integrates the core themes including TME remodeling, immunotherapy-related skeletal muscle injury mechanisms, inflammatory signaling pathway regulation, and metabolic disorders. Priority is given to including research findings that can reveal the interaction mechanisms between immunotherapy and skeletal muscle dysfunction as well as their clinical translational significance. Therefore, we propose TME-targeted strategies for muscle protection. By integrating multidisciplinary approaches, this review aims to provide a scientific basis for enhancing cancer immunotherapy while minimizing skeletal muscle toxicity, thereby advancing cancer treatment toward greater precision and comprehensiveness (17).

## 2 Clinical context and conceptual foundations

### 2.1 Epidemiology and clinical implications

Cancer cachexia is a tumor-driven systemic metabolic-inflammatory syndrome characterized by progressive skeletal muscle loss. Its diagnostic criteria typically include a weight loss of ≥5% within 6 months, or a weight loss of ≥2% in patients with a body mass index (BMI) below 20 kg/m², which is irreversible with conventional nutritional support. As one of the most prevalent complications of malignant tumors, cachexia affects 49.2% of all cancer patients overall, with even higher incidences in solid tumors such as pancreatic cancer, gastric cancer, and lung cancer (74%, 61%, and 63%, respectively) (18). Data from the Chinese population indicate that the incidences of cachexia in pancreatic cancer, gastric cancer, and esophageal cancer are 62.8%, 56.4%, and 51.8%, respectively, representing key challenges in clinical diagnosis and management (19).

Cachexia not only severely impairs patients’ quality of life but also has a clear independent prognostic value. Patients with cachexia have a 41% increased risk of all-cause mortality, along with a significantly reduced chemotherapy completion rate and an elevated risk of treatment discontinuation. Studies have demonstrated that 20%–30% of cancer-related deaths are directly attributed to cachexia and its induced multiple organ failure, rather than the progression of the primary tumor (20). Notably, as the core phenotype of cachexia, the severity of skeletal muscle loss is negatively correlated with treatment tolerance and survival time; in obese populations, muscle loss is often masked by body weight, resulting in “occult sarcopenia” and leading to delayed diagnosis and inadequate intervention.

The widespread clinical application of immune checkpoint inhibitors (ICIs) has revolutionized the therapeutic landscape of malignant tumors while concomitantly altering the clinical spectrum of cachexia and skeletal muscle injury. In the era of immunotherapy, skeletal muscle injury exhibits a dual pathogenic pattern. First, baseline sarcopenia affects up to 61.2% of advanced cancer patients initiating immunotherapy; these patients face a 2.635-fold higher risk of developing any-grade immune-related adverse events (irAEs) and exhibit significantly poorer prognoses (21). Second, immunotherapy can directly induce skeletal muscle injury via irAEs, specifically referred to as ICI-associated myositis. A meta-analysis encompassing 18 phase III randomized controlled trials (RCTs) with a total of 6,838 patients demonstrated that the overall incidence of ICI-associated myositis is approximately 0.38%, which increases to 1%–3% in patients receiving PD-1/PD-L1 inhibitor monotherapy. Despite its relatively low incidence, this specific myositis poses substantial clinical risks: the mortality rate of isolated ICI-associated myositis ranges from 10% to 15%, and it can soar to 40%–50% when complicated by myocarditis or myasthenia gravis (22).

The aforementioned epidemiological characteristics highlight the clinical urgency of the research topic addressed in this study. While immunotherapy may confer survival benefits to patients by reversing the tumor immune microenvironment, it may concurrently accelerate skeletal muscle loss and cachexia progression by inducing immune-related myositis or exacerbating systemic inflammation. This paradox between therapeutic benefits and muscle injury renders the investigation of skeletal muscle injury mechanisms and the development of intervention strategies in the context of immunotherapy an urgent clinical and scientific problem that requires immediate resolution.

### 2.2 Conceptual framework and scope of this study

To ensure the consistency of concept usage and logical coherence throughout the manuscript, several key terms are operationally defined herein. “Tumor microenvironment (TME)” specifically refers to a dynamic cellular and molecular ecosystem consisting of tumor cells, immune cells, stromal cells, extracellular matrix, and various soluble molecules, which exerts a central regulatory role in tumor progression and treatment response. “Immunotherapy” primarily encompasses immune checkpoint inhibitors (ICIs), adoptive cell therapy (ACT, including CAR-T and TCR-T), and cytokine-based therapeutic strategies; unless otherwise specified, it does not include other immunomodulatory modalities. “Skeletal muscle injury” denotes direct or indirect abnormalities in skeletal muscle structure, metabolism, and function that arise in the context of systemic immune activation and TME remodeling induced by immunotherapy, including immune-related myositis, metabolic dysfunction, impaired regenerative capacity, and cachexia-associated muscle wasting. To avoid an overly broad interpretation of “skeletal muscle dysfunction,” this study specifically defines it as a composite of structural and functional abnormalities, including reduced skeletal muscle mass, disruption of myofiber structure, energy metabolism disturbances, and declines in muscle strength or endurance. In the context of immunotherapy, these alterations may stem not only from systemic inflammation and metabolic reprogramming but also manifest as immune-related myopathy, an immunotherapy-induced autoimmune muscle injury encompassing immune-related myositis, myasthenia gravis-like syndrome, and other inflammatory myopathy spectra.

“Immune cell infiltration” as described in this manuscript specifically refers to the pathological process whereby effector immune cells (e.g., CD8^+^ T cells and macrophages) break through tissue barriers, migrate directionally, and accumulate abnormally within skeletal muscle tissue upon immunotherapy activation, rather than merely referring to immune cell infiltration within tumor tissue. By clarifying these concepts, this manuscript distinguishes immunotherapy-related muscle toxicity from pre-existing sarcopenia or tumor-driven cachexia, while also highlighting the crosstalk and overlap at the mechanistic level. By clarifying these concepts, this study distinguishes immunotherapy-related muscle toxicity from pre-existing sarcopenia or tumor-driven cachexia. Sarcopenia primarily refers to the progressive decline in skeletal muscle mass and function associated with aging or chronic disease, with mechanisms centered on neuromuscular degenerative changes and metabolic abnormalities. Cachexia, conversely, is a systemic metabolic syndrome driven by malignancy or chronic inflammation, characterized by persistent muscle wasting, weight loss, and elevated inflammatory cytokines, which is typically refractory to reversal through nutritional support alone. While immunotherapy-related muscle injury may partially overlap phenotypically with these conditions, its core mechanisms are more closely linked to immune overactivation, cytokine release, and immune cell-mediated tissue damage, potentially exhibiting crosstalk and synergy with cachexia-related inflammatory pathways.

**Figure 1:**
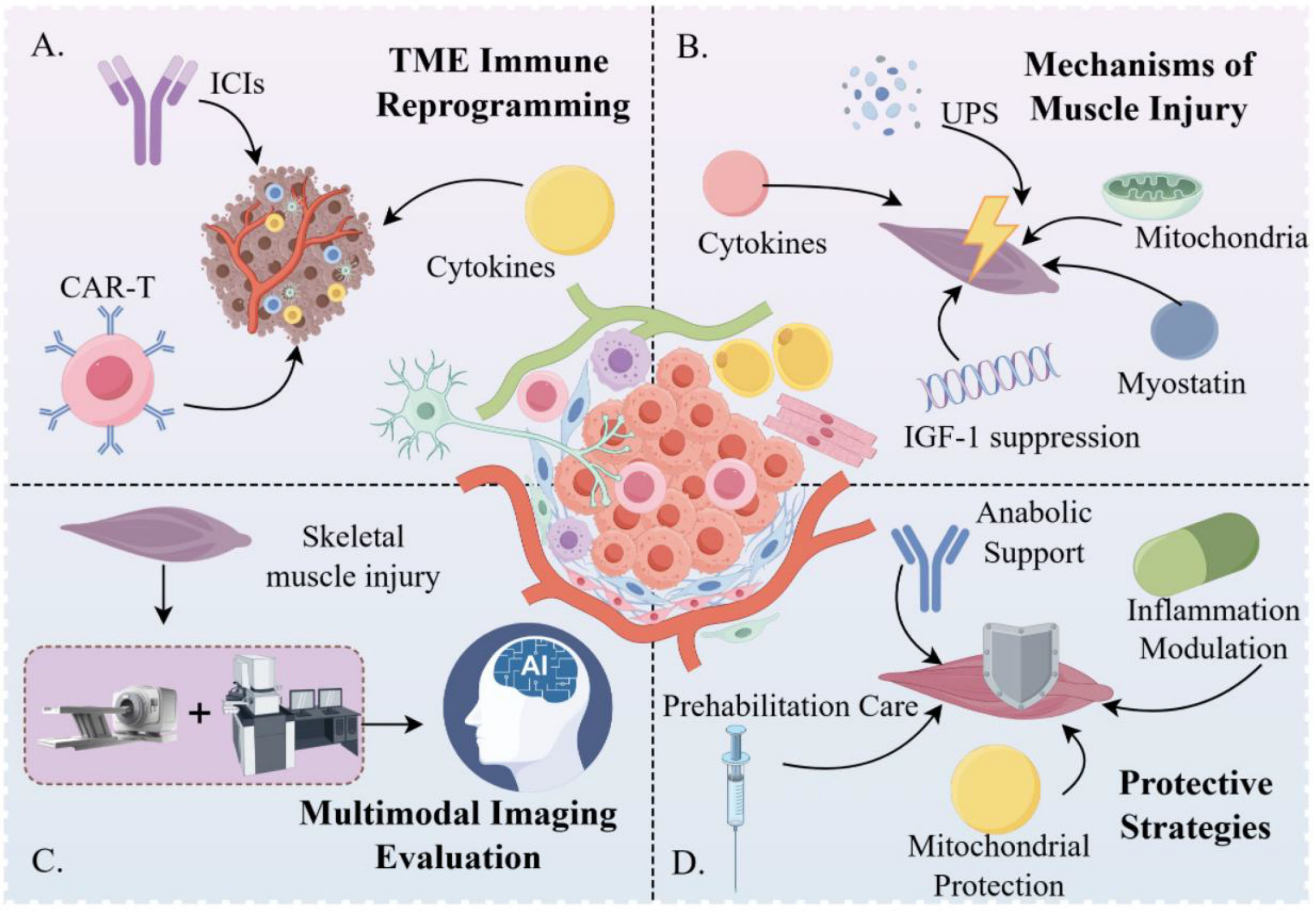
Conceptual framework of immunotherapy-associated skeletal muscle dysfunction and precision intervention strategies. **(A)** Immunotherapy reshapes the ecological architecture of the tumor microenvironment; **(B)** Immunotherapy-driven metabolic disequilibrium and functional decline in skeletal muscle; **(C)** Advanced imaging modalities enable phenotypic stratification and risk prediction; **(D)** Multidimensional therapeutic strategies coordinate toxicity mitigation with preservation of antitumor efficacy.

This manuscript focuses on the bidirectional crosstalk between dynamic changes in the TME regulated by immunotherapy and pathological alterations in skeletal muscle. It prioritizes analyzing how mechanisms, including immune activation, cytokine signaling, metabolic reprogramming, abnormal angiogenesis, and stromal-immune cell crosstalk, collectively mediate skeletal muscle dysfunction. Meanwhile, in the context of surgical intervention, this manuscript explores its clinical translational significance by integrating the characteristics of perioperative metabolic stress and immune regulation. Structurally, this manuscript first systematically summarizes the compositional and functional remodeling features of the TME under different immunotherapy modalities; second, it deciphers how immune activation and TME alterations synergistically act on skeletal muscle tissue from the perspectives of inflammatory factor spillover, immune cell infiltration, metabolic-vascular imbalance, and regeneration inhibition; finally, it integrates multimodal imaging assessment tools and multidimensional protective strategies, and proposes a translational model for decoupling antitumor efficacy from skeletal muscle toxicity. The overall logical framework of this review is illustrated in **Figure 1**. The figure is structured into four core components: immunotherapy-driven immune remodeling of the TME, key molecular and metabolic mechanisms underlying skeletal muscle injury, advanced imaging technologies for auxiliary diagnosis, and integrated protective and interventional strategies, thereby establishing a coherent research pathway that bridges mechanistic insights with clinical translation.

## 3 Composition, Characteristics, and dynamic remodeling of the TME in immunotherapy

### 3.1 Composition and characteristics of the TME

The TME is a complex cellular ecosystem consisting of tumor cells, immune cells, and stromal cells (6). Tumor cells drive TME remodeling through genetic mutations and metabolic reprogramming to sustain rapid proliferation (23). Immune cells play a dual role: CD8^+^ T cells and NK cells mediate tumor cytotoxicity, while myeloid-derived suppressor cells (MDSCs) and tumor-associated macrophages (TAMs) promote immune evasion via immunosuppressive cytokines (e.g., IL-10, TGF-β) (24). Stromal cells, particularly cancer-associated fibroblasts (CAFs), further shape the TME by secreting extracellular matrix (ECM) components and pro-tumor cytokines (TGF-β, PDGF) (25). A defining characteristic of the TME is the paradoxical coexistence of immunosuppressive and immunoreactive components. Tumor metabolic reprogramming creates an immunosuppressive barrier that impairs immune cell function, thereby limiting the efficacy of immunotherapy (16).

### 3.2 Regulatory mechanisms of immunotherapy within the TME inhibition of immune checkpoints (PD-1/PD-L1 and CTLA-4)

**Figure 2:**
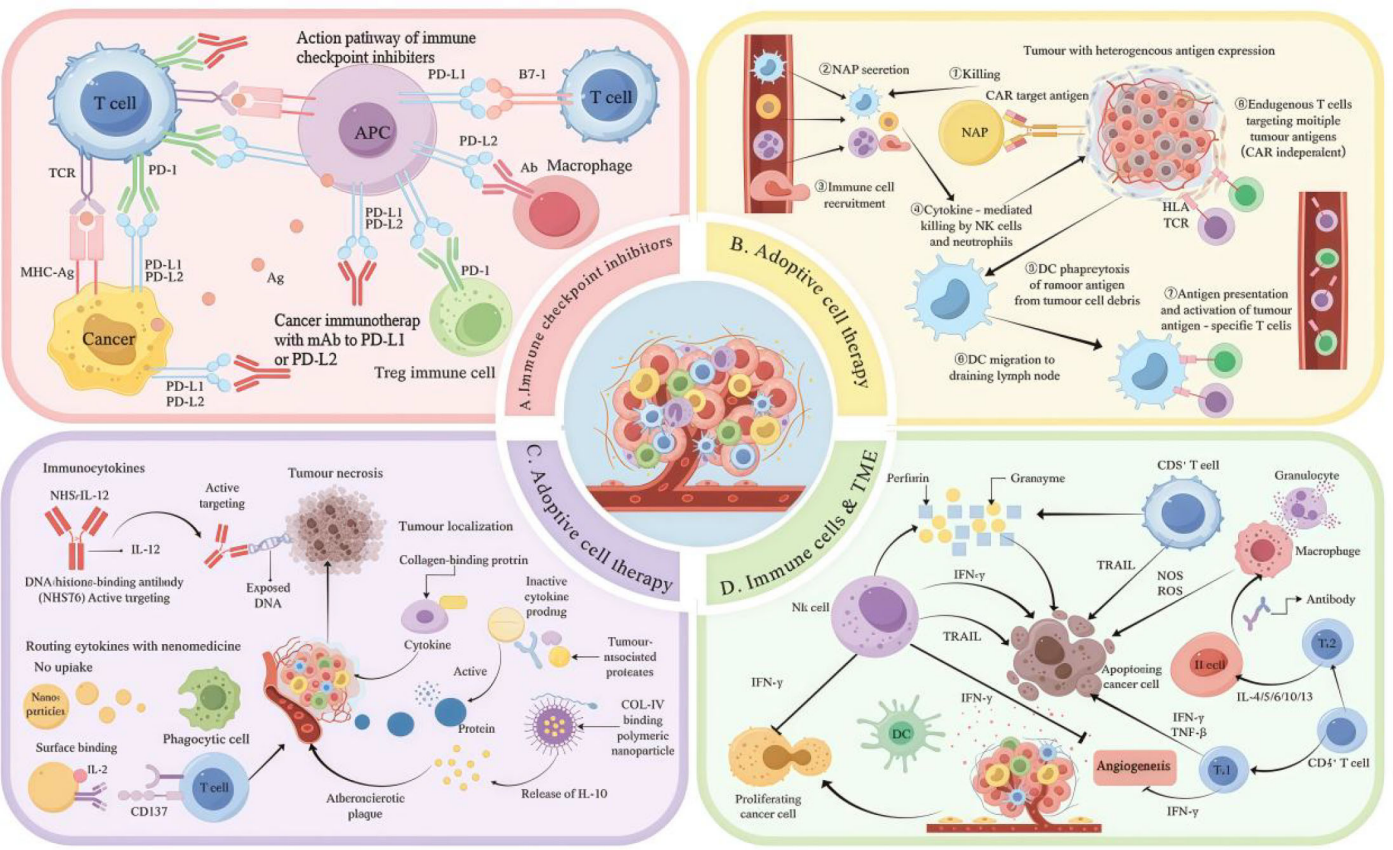
Regulatory mechanisms of immunotherapy within the TME: **(A)** Immune checkpoint inhibitors activate anti-tumor immune responses; **(B)** ACT enhances anti-tumor immunity; **(C)** Cytokines reshape the composition of immune cell populations; **(D)** Synergistic immunosuppression mediated by immune cells and the TME.

ICIs reactivate the body’s anti-tumor immunity by blocking aberrant interactions of PD-1/PD-L1 and CTLA-4 (26), whose core function is to relieve TME-mediated suppression of cytotoxic T cells and restore their tumor-killing capacity (27). As shown in **Figure 2A, ICIs** exert effects via key signaling pathways in the interaction network of T cells, tumor cells and antigen-presenting cells (APCs) (28). Specifically, PD-1/PD-L1 inhibitors disrupt the binding of tumor cell PD-L1 to T cell PD-1, alleviating the inhibition of cytotoxic T cells and promoting the proliferation of tumor-infiltrating lymphocytes (29). CTLA-4 inhibitors block the binding of Treg-expressed CTLA-4 to APC surface CD80/CD86, reducing Treg-mediated immunosuppression and enhancing effector T cell costimulatory signaling (30). The “dual checkpoint blockade” synergistically reprograms TME immune balance to boost anti-tumor immunity and inhibit tumor proliferation (31). In PD-L1-high tumors, PD-1/PD-L1 inhibitors reverse M2 macrophage polarization, reduce IL-10 secretion and promote APC maturation (32), forming a “dual-release inhibition” effect where the PD-1/PD-L1 axis enhances effector T cell activity and the CTLA-4 axis reduces immunosuppressive cell infiltration.

### 3.3 Infiltration and activation of the TME by ACTs (CAR-T and TCR-T)

ACT is an innovative cancer immunotherapy that involves ex vivo modification and expansion of a patient’s immune cells (mainly T cells), which are reinfused to enhance the immune system’s tumor-recognition and cytotoxic capacity (33). As shown in **Figure 2B, CAR**-T and TCR-T, the two major ACT modalities, mediate distinct infiltration and activation in the TME (34). CAR-T cells degrade ECM physical barriers (e.g., hyaluronic acid, type I collagen) and express chemokine receptors (e.g., CCR5) to respond to tumor-secreted chemokines (e.g., CCL5), thereby promoting intratumoral migration and infiltration (35). In contrast, TCR-T cells are activated in an MHC-dependent manner, secrete IFN-γ to recruit NK cells, and target heterogeneous tumor-associated antigens via neutrophil-activating protein secretion and immune cell recruitment to exert anti-tumor effects (36). IFN-γ secreted by TCR-T cells also downregulates tumor cell PD-L1 expression, reducing immune evasion and enhancing NK cell-mediated cytotoxicity (37). Due to the inhibitory TME of solid tumors (e.g., high PD-L1 expression), ACT is often combined with PD-1/PD-L1 inhibitors to relieve T cell suppression and boost TCR-T anti-tumor activity (38). Overall, ACT remodels the TME through immune cell recruitment, activation and direct tumor killing, inducing a coordinated and robust anti-tumor immune response (39).

### 3.4 Modulation of immune cells by cytokine therapies (IL-2, IL-12, and IFN-γ)

Cytokine therapy is a treatment option that utilizes cytokines to regulate the immune system and enhance the body’s immune response to cancers and other diseases (40). Its primary function is to modify the immune cell composition within the TME by affecting the proliferation, differentiation, activation, and functionality of immune cells, thereby enhancing anti-tumor immunity (41). **Figure 2C** shows that cytokines are essential in the TME as they interact with immune and tumor cells, forming a complex immunological regulatory network. In ACT, cytokines, including IL-12, are employed using targeted delivery platforms, such as NHS-IL-12 conjugated with DNA nanocarriers and tumor-targeting antibodies (NHS), to enhance tumor site localization (42). This process involves collagen-binding proteins in the TME (43). **Figure 2C** also depicts the mechanisms associated with tumor necrosis: circulating cytokines bind to nanoparticles, with “no uptake” regions labeled accordingly (44). These nanoparticles interact with phagocytic cells through surface tags (CT137 labeling), prompting phagocytes to assume alternative phenotypes for cytokine processing. During this process, inert cytokine precursors are activated by tumor-associated proteases, resulting in the release of active cytokines including IL-12. Additionally, nanoparticles bearing CD-IV–binding peptides participate in the regulated release of IL-12, further strengthening anti-tumor immune responses (45).

### 3.5 Synergistic immunosuppression between immune cells (CD8^+^ T Cells, NK cells, and dendritic cells) and the TME

Immune cells are key regulators in the TME, mediating tumor growth and progression via diverse mechanisms (46), with dynamic crosstalk among core subpopulations illustrated in **Figure 2D**. CD8^+^ T cells kill tumor cells directly by releasing perforin and granzyme after recognizing tumor-associated antigens via T cell receptors (47). NK cells exert cytotoxicity through granzyme and perforin, induce tumor cell apoptosis via the FasL-Fas pathway, and secrete IFN-γ and TNF-α to activate other immune cells (48). Macrophages polarize into M1 phenotypes that phagocytose tumor debris and secrete proinflammatory cytokines, while M2-type TAMs secrete IL-10 and other immunosuppressive factors to promote tumor immune evasion (49). Mature DCs prime anti-tumor immunity by presenting tumor antigens to T cells, guiding T cell migration and secreting IL-12 to drive Th1 cell differentiation (50). Tregs suppress immune responses through direct cell contact and secretion of IL-10 and TGF-β (51), while B cells enhance anti-tumor immunity by producing tumor-specific antibodies, mediating phagocytic clearance, activating the complement system and boosting NK cell cytotoxicity (52). Under immune pressure, surviving tumor cells upregulate immunosuppressive molecules (e.g., PD-L1) and secrete TGF-β/IL-10 to remodel the TME for immune evasion (53). These immune cell subpopulations act as bidirectional modulators of tumor progression, collectively maintaining TME immune balance and serving as critical therapeutic targets for cancer immunotherapy (54).

## 4 Mechanisms of skeletal muscle injury induced by immunotherapy

### 4.1 Spillover mechanisms of inflammatory cytokines (TNF-α and IL-6)

**Figure 3:**
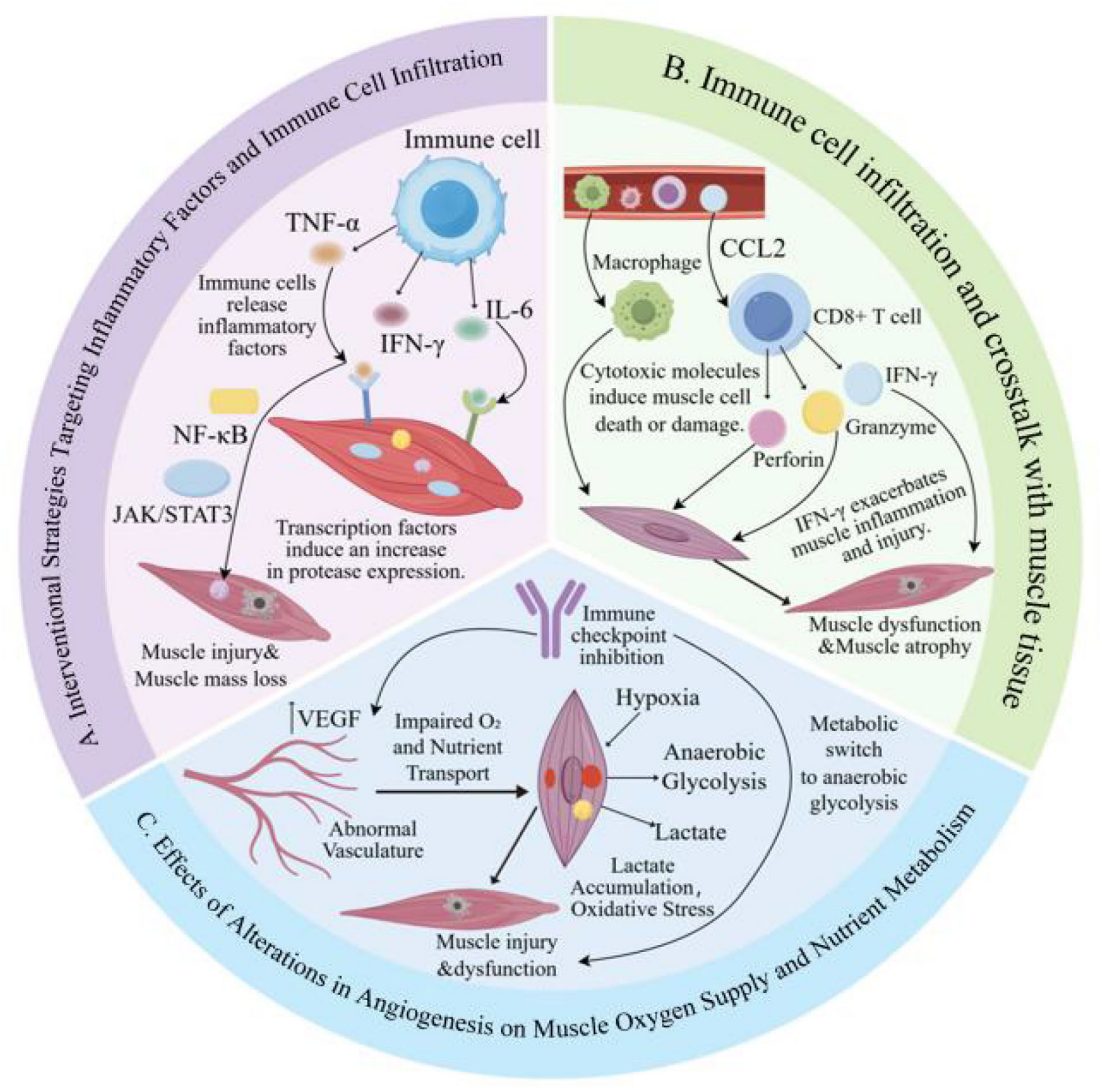
Multidimensional Mechanisms of Skeletal Muscle Injury Induced by Immunotherapy. **(A)** Muscle injury induced by inflammatory cytokines secreted by immune cells. **(B)** Muscle injury induced by cytotoxic molecules released by immune cells. **(C)** Metabolic dysfunction in the muscle tissue triggered by aberrant angiogenesis.

During immunotherapy, immune cells secrete excessive levels of inflammatory cytokines, which activate intracellular signaling pathways within muscle cells. This activation results in the upregulation of transcription factors and a subsequent increase in protease expression, leading to muscle damage and mass loss (55). This phenomenon is observed in multiple pathological conditions including acute inflammation, cancer-related cachexia, and chronic inflammatory diseases. **Figure 3A** shows the infiltration of immune cells and the damaging effects of inflammatory cytokines on muscle tissue (56). In regions of muscle injury and inflammation, immune cells, including macrophages and CD8^+^ T cells, secrete various inflammatory cytokines, including TNF-α, IL-6, and IFN-γ (57). Different immunotherapeutic subtypes can activate the NF-κB and JAK/STAT3 pathways in a subtype-specific manner: ICIs primarily enhance cytokine secretion by CD8^+^ T cells and macrophages to indirectly activate these pathways, adoptive cell therapy (CAR-T/TCR-T) directly amplifies pathway activation effects via intracellular signal transduction, and cytokine therapy initiates pathway cascades by directly binding exogenous cytokines to myocyte receptors (58). Specifically, upon binding of tumor necrosis factor-α (TNF-α) to myocyte TNF receptors (TNFR1/TNFR2), the downstream TRAF6-RIP1-IKK complex is activated, thereby promoting the phosphorylation and degradation of IκBα; this enables the nuclear NF-κB (p65/p50 heterodimer) to be released and initiate transcription. In contrast, interleukin-6 (IL-6) binds to the gp130/IL-6R receptor complex, activating JAK1/JAK2 kinases, which further mediate the tyrosine phosphorylation and dimerization of STAT3; the activated STAT3 then translocates into the nucleus to drive target gene expression (59). Activated NF-κB and STAT3 can transcriptionally upregulate the ubiquitin-proteasome system (Atrogin-1, MuRF-1) and caspase-related genes (Caspase-3, Caspase-9) in myocytes. On one hand, this mediates muscle protein degradation; on the other hand, it triggers endogenous apoptotic signals, thereby collectively inducing myocyte damage through two distinct mechanisms: protein catabolism and cell death (60).

Increased proteolytic activity deteriorates muscle cell constituents, leading to muscle cell damage and loss of muscle mass (61). Additionally, **Figure 3** demonstrates that immune cells can induce muscle apoptosis or damage by releasing cytotoxic molecules including perforin and granzyme. The combined effects of immune cell infiltration and cytokine release lead to muscle dysfunction and atrophy (62). IL-6 and TNF-α synergistically activate the NF-κB and JAK/STAT3 signaling pathways, markedly enhancing muscle protein degradation and loss of muscle mass (63). This mechanism is prevalent in multiple pathological contexts and may represent a promising therapeutic target for the treatment of muscle-wasting diseases (64).

In summary, the massive secretion of pro-inflammatory cytokines such as TNF-α and IL-6 induced by immunotherapy directly drives skeletal muscle protein degradation and apoptosis through the activation of NF-κB and JAK/STAT3 signaling pathways. However, this linear injury model based on cytokine spillover faces significant complexities in clinical translation and should not be viewed simply as the sole determinant of muscle destruction. Existing research predominantly focuses on characterizing systemic inflammation, often overlooking the spatial heterogeneity between elevated systemic cytokine levels and the actual severity of local muscle damage. This disconnect suggests that circulating cytokine spillover may merely serve as an initiating trigger; the ultimate progression to severe muscle toxicity likely depends on how these systemic signals synergize with metabolic stress or satellite cell regenerative impairment within the local skeletal muscle microenvironment. Furthermore, clinical strategies aimed at mitigating muscle injury solely through systemic blockade of these inflammatory pathways inevitably carry the risk of compromising anti-tumor immunosurveillance. Therefore, future mechanistic insights should move beyond merely cataloging systemic inflammatory networks. Instead, research should explore the mechanistic decoupling of anti-tumor efficacy from tissue damage by targeting muscle-specific downstream effectors, such as local E3 ubiquitin ligases to precisely intercept collateral skeletal muscle damage while preserving the therapeutic potency of immunotherapy.

### 4.2 Infiltration mechanisms of immune cells (CD8^+^ T cells and macrophages)

During immunotherapy, immune cell infiltration is an essential mechanism contributing to skeletal muscle injury, particularly involving CD8^+^ T cells and macrophages. In this context, immune cell infiltration refers specifically to the pathological process in which immunotherapy-activated immune cells breach tissue barriers and undergo directed migration into skeletal muscle tissue. **Figure 3B** illustrates the complex interplay between immune cell infiltration and muscle-tissue injury. Guided by chemokines, including CCL2 and CD8 + T cells, macrophages migrate into muscle tissue. CD8 + T cells release perforin and granzyme. Perforin compromises the muscle cell membrane, causing leakage of intracellular content and osmotic imbalance, thereby accelerating necrosis. Granzyme infiltrates the cell through perforin-formed pores, initiating caspase cascades and inducing apoptosis in muscle cells (65). Simultaneously, macrophages release proinflammatory mediators, including TNF-α and IL-1β. Combined with IFN-γ, these cytokines trigger an inflammatory cascade that enhances the local immune responses. IFN-γ upregulates MHC class I molecules on the surface of muscle cells, increasing their visibility to CD8^+^ T cells and amplifying immune-mediated cytotoxicity (66). Moreover, IFN-γ activates the JAK/STAT signaling pathway to modulate AIM2 inflammasome activation, facilitating pyroptosis in mononuclear cells and enhancing inflammatory responses (67). TNF-α further facilitates muscle degradation by stimulating the NF-κB pathway, which enhances the transcription of ubiquitin ligase genes and increases protease synthesis, thereby accelerating muscle protein catabolism through the ubiquitin-proteasome system (68). These immune-mediated mechanisms collectively compromise the muscle architecture, resulting in muscular dysfunction, atrophy, and mass loss, resulting in impaired muscle contractility and progressive wasting (69).

In summary, the pathological infiltration of CD8^+^ T cells and macrophages triggered by immunotherapy constitutes the core driver of skeletal muscle injury. Through the perforin-granzyme system and local release of proinflammatory cytokines, they directly mediate myocyte necrosis, apoptosis and protein degradation. However, existing mechanistic studies are mostly limited to the description of the destructive effects following immune cell infiltration, and fail to deeply reveal the underlying logic of how immune cells break through skeletal muscle immune homeostasis and are specifically recruited to non-tumor tissues. Simply attributing this process to chemokine-mediated passive migration overlooks the critical shift in the perception of immune signals by the skeletal muscle microenvironment after immune checkpoint blockade, as well as the potential molecular mimicry or cross-reactivity between tumor antigens and muscle antigens. This disconnect at the research level makes it clinically difficult to distinguish between the effective infiltration required for anti-tumor efficacy and the pathological infiltration that causes tissue damage. Therefore, future mechanistic analyses must move beyond the mere cataloging of infiltration phenomena and toward an in-depth assessment of the specificity of immune recruitment, with a focus on exploring how to block the specific immune attack against skeletal muscle while preserving systemic immunotherapeutic efficacy by precisely regulating local chemokine gradients or maintaining the immune barrier function of muscle fibers.

### 4.3 Mechanisms of aberrant angiogenesis

Alterations in angiogenesis during immunotherapy are a significant mechanism underlying skeletal muscle injury. Immunotherapeutic interventions can interfere with the regulation of angiogenic factors, compromising the normal development and function of blood vessels, which significantly affects oxygen and nutrient delivery to muscle tissues (70). **Figure 3C** illustrates how abnormal angiogenesis compromises oxygen supply and metabolic balance in the muscle, resulting in tissue damage. As referred to in this manuscript, “abnormal vascular structure” specifically denotes a pathological vascular state characterized by structural disorganization, impaired endothelial function, and reduced microcirculatory perfusion efficiency, which is formed by abnormal angiogenesis, rather than merely referring to simple changes in vascular morphology. Abnormal vasculature results in structural and functional deficiencies in the blood vessels, particularly impairing vascular endothelial growth factor (VEGF)-mediated oxygen and nutrient delivery. Therefore, muscle cells lack sufficient oxygen and essential nutrients, resulting in hypoxic conditions (71). During hypoxia, muscle metabolism transitions from oxidative phosphorylation to anaerobic glycolysis, leading to lactic acid accumulation (72). This metabolic shift induces oxidative stress, which damages the muscle cells and causes structural injury and functional impairment. The entire process highlights how disrupted angiogenesis due to impaired oxygenation and nutrient transport causes metabolic imbalance and subsequent muscle dysfunction. Furthermore, the hypoxic microenvironment may activate hypoxia-inducible factor 1-alpha (HIF-1α), which upregulates glycolysis-related enzyme expression and promotes the secretion of inflammatory cytokines, including TNF-α. This establishes a vicious cycle of hypoxia, metabolic dysregulation, and inflammation, consequently accelerating muscle tissue damage and functional decline (73).

### 4.4 Synergistic amplification mechanism of immunosuppression and cytokine signaling

Interactions between the immunosuppressive microenvironment of TME and therapy-activated cytokines during immunotherapy synergistically contribute to skeletal muscle damage. Tregs and M2-type TAMs in the TME interact dynamically with effector T cells stimulated by immunotherapy, forming an imbalance between immunosuppression and activation (74). This process is core-regulated by non-coding RNAs (ncRNAs). For instance, specific long non-coding RNAs (lncRNAs) highly expressed in muscle tissue can act as competing endogenous RNAs (ceRNAs) to sequester miR-155, abrogating its translational repression of the Treg chemokine receptor CCR4 and thereby promoting the directional migration and infiltration of Tregs into skeletal muscle tissue. Concurrently, certain microRNAs (miRNAs) can form a post-transcriptional regulatory network by targeting and silencing MyoD, a key myogenic differentiation factor in satellite cells, or directly inhibiting SOCS3, a negative regulator of the JAK/STAT3 pathway. These miRNAs synergize with cytokines secreted by Tregs to exacerbate muscle regeneration impairment and protein degradation (75). Cytokines secreted in this context exhibit synergistic amplification effects that induce muscle injury. As referred to in this manuscript, the “synergistic amplification effect” refers to a nonlinear enhancement mechanism in which multiple inflammatory and immune regulatory signaling pathways, through mutual promotion and positive feedback, produce an effect that exceeds the additive effect of individual cytokines. Abnormal vasculature results in structural and functional deficiencies in the blood Simultaneously, Tregs secrete IL-10 and TGF-β, which directly inhibit satellite cell proliferation and muscle fiber regeneration, adversely affecting the skeletal muscle. Clinical studies have demonstrated a positive correlation between Treg infiltration in muscle tissue and reduction in muscle fiber cross-sectional area in patients treated with PD-1 inhibitors (r = 0.68, *P <* 0.01) (76). Furthermore, increased IL-6 and colony-stimulating factor 1 levels in the TME induce TAM polarization toward the M2 phenotype. M2-type TAMs release IL-10, which activates the JAK/STAT3 pathway in muscle cells and upregulates E3 ubiquitin ligase MuRF-1 expression, thereby accelerating the degradation of myosin heavy chains (77). Although immunotherapy can partially reverse M2 polarization, residual M2-type TAMs, combined with IFN-γ released by effector T cells, create a “proinflammatory versus anti-regenerative” paradox: IFN-γ enhances MHC class I expression, promoting dysregulated T cell-mediated assault on muscle tissue, while persistent TGF-β release from M2 TAMs impedes the differentiation of myogenic progenitor cells (78).

### 4.5 Mechanisms of metabolic reprogramming and vascular dysfunction

Metabolic reprogramming in the TME combined with immunotherapy-induced vascular impairment synergistically leads to skeletal muscle damage. These intertwined processes trigger a cascade of complex pathophysiological events that severely impair energy metabolism and muscle function. Tumor cells are essential for this pathology because of their distinct metabolic profiles (79). Owing to the Warburg effect, tumor cells preferentially produce large quantities of lactate through aerobic glycolysis, thereby reducing the pH of the TME to approximately 6.0–6.5. Lactate is transported through monocarboxylate transporter 4 and diffuses into the systemic circulation, reaching skeletal muscle tissues (80). Upon entering muscle cells, lactate inhibits mitochondrial complex I activity, leading to a 30%–40% decrease in ATP synthesis and triggering a burst of reactive oxygen species (ROS). ROS damage calcium channels on muscle fiber membranes (ryanodine receptors), disrupt calcium homeostasis, and directly reduce contractile efficiency. Immunotherapy exacerbates muscle damage by disrupting angiogenesis (81). However, PD-1/PD-L1 inhibitors suppress the VEGF-A/VEGFR2 signaling pathway, decreasing microvascular density in muscle tissue and extending oxygen diffusion times by 2–3-fold compared with normal tissue. Conversely, CTLA-4 blockade activates T cells to release IFN-γ, which induces endothelial cells to produce adhesion molecules including ICAM-1, thereby facilitating microvascular constriction and thrombosis. These synergistic effects drive skeletal muscle cells into a state of dual deficiency, both oxygen- and nutrient-deprived, activating HIF-1α. Driven by this metabolic stress, myocytes undergo extensive metabolic reprogramming: lactate post-transcriptionally represses peroxisome proliferator-activated receptor gamma coactivator 1-alpha (PGC-1α), thereby systemically downregulating the expression of genes involved in mitochondrial biogenesis and oxidative phosphorylation. In parallel, ketone bodies activate the AMP-activated protein kinase (AMPK) pathway, and these two metabolic cues collectively drive the transition of myocytes toward a glycolytic phenotype (82). Critically, this metabolic dysregulation also establishes a bidirectional metabolic crosstalk network between skeletal muscle and the TME. Enhanced glycolysis in myocytes results in the excessive release of pyruvate and free fatty acids; upon entering the TME, these metabolites not only serve as alternative energy substrates to fuel tumor cell proliferation but also upregulate the expression of key glycolytic enzymes in tumor cells, thereby substantially augmenting their metabolic activity and proliferative potential. Activated hypoxia-inducible factor 1-alpha (HIF-1α) subsequently acts in synergy with nuclear factor-kappa B (NF-κB) to further upregulate the expression of the glycolytic enzyme lactate dehydrogenase A (LDH-A) and the ubiquitin ligase atrogin-1, thus establishing a positive feedback loop that links metabolic dysregulation to accelerated protein degradation (83). This vicious cycle perpetually amplifies muscle damage, ultimately leading to progressive functional impairment of skeletal muscle (84).

In summary, tumor-derived lactate accumulation and immunotherapy-induced microvascular impairment jointly drive metabolic reprogramming and functional failure in skeletal muscle, forming a malignant feedback loop between metabolic stress and protein degradation. However, this complex metabolic profile should not be simplified as a linear accumulation of biochemical reactions; it essentially reflects the severe nutritional competition and resource sequestration between tumors and the host. Current mechanistic interpretations mostly focus on describing the unidirectional damage of metabolic products to muscle, while neglecting the systemic role of skeletal muscle, as the body’s largest metabolic reservoir, in passively delivering energy substrates to tumors under the pressure of immunotherapy. The profound contradiction of this metabolic shift lies in the fact that simply alleviating toxicity by enhancing muscle metabolism may inadvertently provide additional nutritional support for tumor growth, thereby resulting in therapeutic antagonism. Therefore, future research must move beyond the superficial description of metabolic pathways and vascular lesions, and shift toward in-depth intervention in the metabolic crosstalk mechanism between tumors and muscle. The focus should be on exploring how to precisely cut off the pathway of tumor energy sequestration from muscle, thus improving the metabolic homeostasis of patients without disrupting the anti-tumor balance of immunotherapy.

### 4.6 Stroma–immune cell crosstalk-mediated suppression of muscle regeneration

The interaction between stromal components and infiltrating immune cells within the TME forms a complex physical and functional barrier, which inhibits skeletal muscle regeneration. However, modifications to the ECM significantly hinder muscle repair. CAFs secrete excessive amounts of type I collagen and fibronectin, which aberrantly aggregates in the muscle interstitium. These ECM components stimulate muscle fibroblasts through integrin α5β1 signaling, facilitating their differentiation into myofibroblasts. The resulting cells secrete profibrotic cytokines, including TGF-β and PDGF, which promote perimyofiber fibrosis and intensify tissue remodeling (85). However, immune cell infiltration can hinder muscle repair. Infiltrating CD8^+^ T cells directly damages muscle fibers through the perforin–granzyme axis. Moreover, M1-polarized macrophages stimulated by immunotherapy may become excessively proinflammatory while clearing necrotic myofibers. These macrophages release matrix metalloproteinases, which degrade ECM components, including laminin and collagen IV, thereby compromising the niche essential for satellite cell adhesion and maintenance. This impairs the proliferation and regenerative capacity (86). Transcriptomic analyses of injured muscle tissue revealed downregulation of key myogenic differentiation genes, including MyoD and Myogenin, along with upregulation of fibrosis-related genes, including Col1a1 and Fn1. These alterations contribute to a vicious cycle of “injury-fibrosis-repair suppression.” ECM remodeling and immune cell infiltration within the TME synergistically impair normal skeletal muscle repair and regeneration through multiple mechanisms, aggravating muscle injury, and hindering functional recovery.

### 4.7 Integrated molecular pathways linking muscle degradation and metabolic reprogramming in cancer immunotherapy

Ubiquitin-Proteasome System: The ubiquitin−proteasome system (UPS) functions as the central execution pathway in immunotherapy−associated skeletal muscle atrophy, and its aberrant activation signifies the formal onset of catabolic programs. Within an inflammation−driven milieu, expression of the E3 ubiquitin ligases MuRF1 and Atrogin−1/MAFbx is persistently elevated. These ligases selectively target critical structural and regulatory proteins, including myosin heavy chain, titin, and myogenic regulatory factors, for K48−linked polyubiquitination and subsequent degradation by the 26S proteasome, directly causing myofibrillar disassembly and reduced muscle mass (87). Distinct from simple nutritional deprivation, UPS activation under immunotherapy displays a pronounced inflammation−dependent signature. At the signaling level, TNF−α and IL−6 activate FoxO transcription factors through NF−κB and STAT3; nuclear translocation of FoxO1/FoxO3 represents a pivotal transcriptional switch governing the upregulation of MuRF1 and Atrogin−1. Concurrently, non−coding RNA networks exert fine−tuned regulation: multiple miRNAs are downregulated during inflammation, thereby relieving repression of FoxO factors or E3 ligases; select lncRNAs potentiate NF−κB activity and sustain catabolic signaling (88). Accordingly, aberrant UPS activation arises not merely as a direct response to isolated inflammatory stimuli, but as a combined outcome of dysregulated transcription and disordered post−transcriptional control.

Pro-inflammatory Cytokine Axis and NF-κB pathway: The proinflammatory cytokine axis acts as the upstream driver system in immunotherapy-related skeletal muscle injury, with TNF−α and IL−6 serving as the key nodal mediators. TNF−α triggers activation of the IKK complex through TNFR1, thereby promoting nuclear translocation of NF−κB p65/p50 and directly driving the transcription of MuRF1 and Atrogin−1. IL−6 amplifies inflammatory signals via the JAK/STAT3 pathway and forms a synergistic regulatory network with NF−κB, leading to sustained enhancement of catabolic gene expression (89). This inflammatory hub not only regulates protein degradation but also exacerbates oxidative stress by inducing iNOS expression and ROS generation, while promoting myocyte apoptosis through upregulation of caspase family genes. Sustained STAT3 activation also represses PGC−1α, a key mitochondrial biogenesis factor, indirectly impairing oxidative metabolic capacity. Notably, negative feedback regulatory mechanisms of inflammation are gradually compromised under chronic immune activation, resulting in persistent NF−κB signaling and the establishment of a chronic catabolic microenvironment.

Myostatin/Activin Pathway: Myostatin and activin A are key negative regulators of skeletal muscle growth and regeneration, and play a central role in mediating regeneration inhibition during immunotherapy-associated muscle wasting. Both factors activate Smad2/3 signaling via the ActRIIB receptor, suppressing the expression of MyoD and Myogenin to impair the proliferation and differentiation capacity of satellite cells. Distinct from mere enhancement of catabolism, this pathway traps skeletal muscle in a state of persistent insufficient repair by blocking its regenerative potential (90). The inflammatory microenvironment upregulates myostatin expression and reinforces catabolic signaling through crosstalk between NF-κB and Smad3. Additionally, other TGF-β family members, such as GDF-15, are upregulated during immunotherapy, which may further perturb muscle metabolism and repair capacity. Non-coding RNAs also participate in this regulatory hierarchy: downregulation of specific microRNAs (miRNAs) relieves their inhibition on myostatin or Smad3, leading to sustained restriction of muscle regeneration. This pathway thus constitutes a critical node mediating the dual insult of enhanced catabolism and impaired regeneration.

The IGF-1/Akt/mTOR Axis: IGF-1/Akt/mTOR axis represents the core anabolic pathway governing skeletal muscle protein synthesis and cell survival. Under physiological conditions, IGF-1 activates PI3K/Akt signaling, which in turn triggers mTORC1-mediated translation, promotes phosphorylation of S6K1 and 4E-BP1, and sustains myofibrillar protein synthesis (91). However, during chronic inflammation induced by immunotherapy, TNF-α and IL-6 suppress IGF-1 signaling and Akt phosphorylation, thereby attenuating mTORC1 activity and reducing the rate of protein synthesis. Importantly, inhibition of Akt signaling leads to the release and nuclear translocation of FoxO transcription factors, which further upregulate MuRF1 and Atrogin-1 expression, establishing a metabolic imbalance characterized by concurrent hypercatabolism and hyposynthesis.Oxidative stress and endoplasmic reticulum stress also contribute to Akt inactivation, preventing skeletal muscle from mounting compensatory anabolic responses under high catabolic pressure (92). Inhibition of this anabolic axis marks the transition of skeletal muscle from homeostatic balance to a state of persistent net protein loss.

Mitochondrial Dysfunction: Mitochondrial dysfunction constitutes a pivotal metabolic underpinning of immunotherapy-associated muscle fatigue and functional impairment. Inflammation-driven NF−κB activation augments reactive oxygen species (ROS) generation, inducing mitochondrial DNA (mtDNA) damage and depolarization of the mitochondrial membrane potential. Concurrently, sustained STAT3 activation represses peroxisome proliferator-activated receptor gamma coactivator 1-alpha (PGC-1α), impairing mitochondrial biogenesis and turnover. Insufficient adenosine triphosphate (ATP) production diminishes muscle contractile efficiency and compromises cellular resilience to metabolic stress.While PINK1/Parkin-mediated mitophagy exerts a cytoprotective role in the early phase, prolonged inflammation elicits excessive mitochondrial clearance, further reducing mitochondrial mass (93). Mitochondria-derived ROS in turn activates NF−κB, forming a self-reinforcing positive feedback loop between inflammation and oxidative stress. Simultaneously, cytochrome c release triggers the caspase-dependent apoptotic cascade, exacerbating myocyte loss.Mitochondrial metabolic collapse is therefore not merely an energy insufficiency, but a central nodal point that amplifies inflammation and precipitates cell death.

### 4.8 Bidirectional regulatory mechanisms between immunotherapy and skeletal muscle dysfunction

A complex bidirectional regulatory crosstalk exists between immunotherapy and skeletal muscle dysfunction, and this mutual interaction directly modulates immunotherapeutic response efficacy and long-term patient outcomes. On one hand, skeletal muscle status serves as a crucial predictive marker for immunotherapeutic efficacy; on the other hand, therapy-related adverse events conversely impair the body’s functional status, thereby compromising overall treatment outcomes.

Sarcopenia serves as a key predictor of unfavorable prognosis in patients treated with ICIs. Clinical studies have shown that patients with low baseline skeletal muscle mass have significantly shorter progression−free survival and overall survival, with an objective response rate over 30% lower than that in patients with normal muscle mass (94). As a major metabolic reservoir and energy−regulating organ, skeletal muscle critically supports immune function. When muscle mass is depleted, whole−body energy metabolism declines, impairing fuel supply to immune cells, especially CD8^+^ T cells, and rendering them prone to metabolic exhaustion, manifested by reduced effector cytokine secretion and diminished proliferative activity.Muscle damage also leads to aberrant elevation of growth differentiation factor 15 (GDF−15), which suppresses T−cell proliferation and impairs antigen presentation by dendritic cells, further dampening antitumor immunity. Conversely, immunotherapy−related adverse events can exacerbate functional decline, with immune−mediated myositis as a prototypical example. Its pathogenesis involves infiltration of CD8^+^ T cells and macrophages into healthy muscle tissue, triggering acute inflammation and myofiber necrosis that directly cause muscle weakness and impaired mobility (95). Nonspecific T−cell invasion into normal muscle also disrupts microenvironmental homeostasis, amplifies local inflammation, and accelerates muscle loss and dysfunction.This treatment−induced functional deterioration not only reduces quality of life but may also compromise antitumor efficacy through treatment interruption or dose modification, ultimately establishing a self−perpetuating detrimental cycle.

The aforementioned pathological changes at the molecular and metabolic levels do not exist in isolation, but rather manifest as characteristic structural alterations, functional decline, and metabolic abnormalities at the macroscopic level. These measurable phenotypes provide clear biological evidence and potential targets for the non-invasive assessment of skeletal muscle injury, while also laying a solid foundation for the establishment of an integrated evaluation system combining imaging and functional assessments.

## 5 Applications of multimodal imaging technologies in the evaluation of skeletal muscle and the TME

### 5.1 Positron emission tomography/computed tomography fusion imaging for evaluating the metabolic microenvironment of skeletal muscle

**Figure 4:**
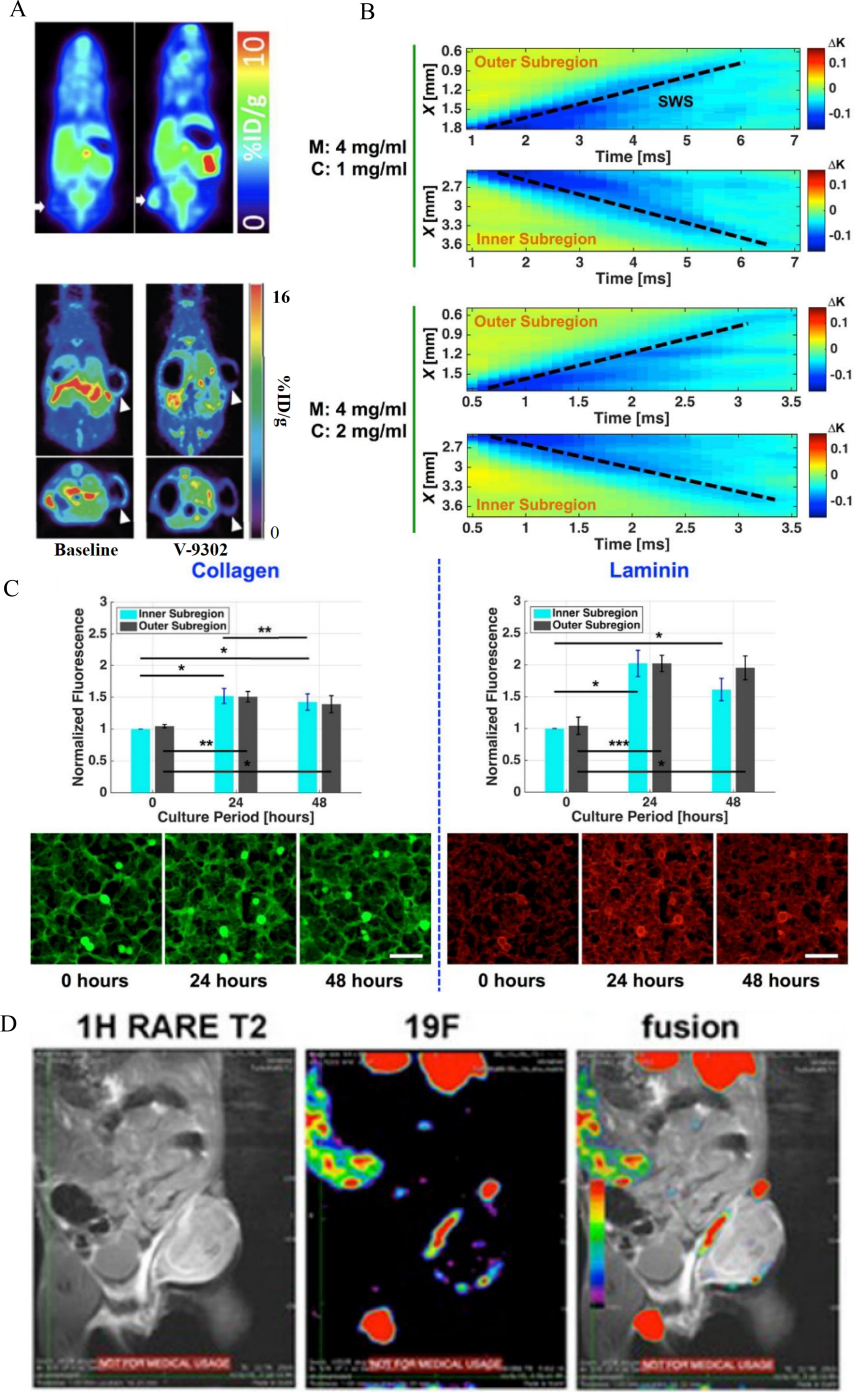
Integrated applications of multimodal imaging technologies in the evaluation of skeletal muscle and its microenvironment. **(A)** PET/CT fusion imaging to assess the metabolic microenvironment of the skeletal muscle (96). **(B)** SWE for evaluating changes in skeletal muscle elasticity (97). **(C)** Fluorescence imaging was used to visualize the distribution of collagen and laminin (97). **(D)** Multimodal imaging allows for comprehensive assessment of the skeletal muscle microenvironment under various treatment conditions (98).

Inflammatory responses and abnormal angiogenesis within the TME substantially affect the metabolic conditions of the skeletal muscles. PET/CT serves as a conduit between metabolic activity and anatomical visualization, facilitating assessment of the metabolic microenvironment by quantifying fluorodeoxyglucose (FDG) uptake in muscle tissue. **Figure 4A** shows that PET/CT fusion imaging employs color-coded gradients to illustrate the metabolic distribution in the skeletal muscle (96). Areas of high FDG uptake indicated active metabolic processes, whereas regions of low uptake reflected suppressed metabolic activity. Research results have demonstrated significant correlations: FDG uptake exhibited a significant positive correlation with levels of the inflammatory marker IL-6 (r = 0.78), indicating that inflammation enhances glucose metabolic demand in muscle tissue. Furthermore, regions exhibiting elevated HIF-1α expression demonstrated significantly increased FDG uptake (*P <* 0.05), indicating that areas of angiogenic dysfunction undergo metabolic reprogramming (99). This imaging modality sensitively detects minor metabolic alterations under inflammatory and angiogenic stress, and offers a visual metabolic basis for diagnosing muscle dysfunction in conditions such as inflammatory myopathies and cancer cachexia. This facilitates the prompt identification of metabolic imbalances and supports the development of targeted intervention strategies (100).

### 5.2 Shear wave elastography for assessing the immune microenvironment of skeletal muscle

The immune microenvironment is a crucial regulator of skeletal muscle homeostasis, with cytokine networks and immune cell infiltration patterns significantly affecting muscle structure and function. SWE has emerged as a powerful tool for visualizing immune microenvironmental changes by precisely measuring the mechanical properties of the muscle tissue. Studies on cancer-associated muscle atrophy have demonstrated that reductions in muscle elasticity are mechanical alterations and mechanistically associated with immune activity, specifically fibrosis mediated by TNF-α and T cell infiltration (**Figure 4B**) (97). In regions with a reduced shear modulus, TNF-α levels were enhanced by 2–3 fold and T cell infiltration was significantly increased (*P <* 0.01). These findings confirm that SWE can non-invasively and dynamically represent immune-mediated remodeling processes within the muscle tissue. In clinical and research settings, SWE can be used to monitor immune-mediated myopathies, including polymyositis and cancer therapy-related myopathies. This technique offers significant insight into the dynamic interplay between immune cells and cytokines in muscle pathology and offers a visual, quantifiable biomarker for assessing the therapeutic efficacy of immunomodulatory interventions (101).

### 5.3 Dynamic contrast-enhanced magnetic resonance imaging for evaluating the vascular microenvironment of skeletal muscle

The angiogenesis balance is crucial for regulating nutrient delivery and functional maintenance in muscle tissues (102). DCE-MRI has become a core modality for assessing the vascular microenvironment through the refined analysis of perfusion parameters. Fluorescence imaging illustrated the time-dependent distribution of ECM components, including collagen and laminin, indicating ongoing vascular remodeling (**Figure 4C**) (97). **Figure 4D** integrates multimodal imaging data to present a composite view of the metabolic, immune, and vascular microenvironmental features across different treatment conditions (98). A comprehensive review and data synthesis resulted in a robust correlation model; blood perfusion parameters derived from DCE-MRI exhibited a significant positive correlation with VEGF expression (r = 0.82). In regions of abnormal angiogenesis, muscle tissue demonstrates a markedly diminished nutrient supply, consequently impairing contractile function and regenerative capacity (103). This imaging technique enables the precise localization of angiogenic abnormalities and provides a functional assessment of muscle integrity, aiding in the risk evaluation of anti-angiogenic therapies (tumor-targeted treatments). DCE-MRI facilitates the optimization of treatment plans and minimizes muscle-related complications associated with vascular inhibition, providing a targeted approach for managing muscle dysfunction associated with vascular microenvironmental disturbances.

However, these imaging technologies were not used for isolation. Multimodal integration offers a more profound understanding of the mechanisms underlying skeletal muscle dysfunction. Multimodal imaging lays the groundwork for precise clinical diagnosis, therapeutic monitoring, and personalized intervention strategies (104).

### 5.4 Precise quantification and integrated analysis system of multimodal imaging assisted by artificial intelligence

Artificial intelligence (AI) technology, leveraging the inherent technical advantages of algorithms such as deep learning and machine learning, has emerged as the core backbone for in-depth interpretation and precise application of multimodal imaging, and its full-process technical framework, spanning feature engineering, model construction, and data fusion, can overcome the inherent technical limitations of manual analysis in skeletal muscle injury assessment, enabling automated quantitative analysis, high-throughput feature mining, and cross-modal heterogeneous data integration for multimodal imaging modalities, including PET/CT, shear wave elastography, and DCE-MRI; as a core subset of AI technology, deep learning relies on the hierarchical feature learning capacity of deep neural networks to sequentially extract and fuse pixel-level, texture-level, and semantic-level features from multimodal imaging, freeing traditional manual analysis from the subjective selection of imaging features and reliance on empirical judgment, thereby establishing an end-to-end correlation analysis framework that links imaging phenotypes to molecular mechanisms (105), and this technical system enables accurate capture of multi-dimensional pathological characteristics of immunotherapy-related skeletal muscle injury, such as metabolic abnormalities, elastic alterations, and vascular perfusion deficits, providing a standardized, quantifiable, and highly reproducible assessment strategy for the early diagnosis, molecular mechanism elucidation, and dynamic monitoring of therapeutic responses in skeletal muscle injury, while through training and optimization using massive imaging datasets, it uncovers subtle features and deep correlation patterns that are undetectable via manual analysis and establishes quantitative association models between imaging features, clinical phenotypes of skeletal muscle injury, and molecular mechanisms, thus laying a robust algorithmic and data-driven foundation for personalized diagnosis and treatment.

Deep learning-based imaging analysis technology constitutes the core technical component for achieving precise multimodal imaging assessment of skeletal muscle injury. The design and optimization of its algorithmic framework directly dictate the accuracy and efficiency of imaging analysis, with a primary focus on two core technical directions: high-precision automatic segmentation of injured regions and in-depth extraction of multi-dimensional radiomic features. Targeted algorithmic modifications have been implemented to accommodate the unique tissue characteristics and pathological features of skeletal muscle imaging (106). For automatic segmentation of injured regions, improved U-Net-based networks (e.g., U-Net++, Attention U-Net) and Mask R-CNN instance segmentation algorithms have addressed key challenges in skeletal muscle imaging—including blurred boundaries of injured regions, irregular lesion morphologies, and the easy oversight of tiny lesions—by integrating skip connections, attention mechanisms, and multi-scale fusion modules; following transfer learning and fine-tuning on skeletal muscle datasets, these algorithms enable pixel-level precise segmentation of PET/CT metabolic lesions, shear wave elastography elastic abnormalities, and DCE-MRI vascular perfusion deficits, with a segmentation accuracy reaching the submillimeter level, thus laying a robust foundation for accurate regional localization in subsequent quantitative analyses. In terms of multi-dimensional feature extraction, deep learning models such as convolutional neural networks (CNNs), Vision Transformers (ViTs), and convolutional autoencoders can automatically extract hundreds of high-throughput radiomic features from multimodal imaging, including texture, morphological, metabolic, elastic, and vascular perfusion features, overcoming the limitation that traditional manual analysis can only capture a dozen or so intuitive features. Meanwhile, effective screening of high-dimensional features is accomplished via feature dimensionality reduction algorithms (e.g., PCA, t-SNE), facilitating the identification of potential imaging biomarkers that are highly correlated with skeletal muscle injury severity, NF-κB/JAK/STAT3 signaling pathway activation status, myofiber apoptosis, and protein degradation levels. This enables non-invasive molecular phenotyping of skeletal muscle injury and completes the indirect mapping from imaging features to underlying molecular mechanisms (107).

Intelligent fusion and correlation analysis of multimodal imaging features constitute the pivotal technical core of artificial intelligence (AI)-assisted systems. Leveraging the cross-modal data fusion capabilities of deep learning, this technology achieves complementary integration of information across diverse imaging modalities, thereby constructing a more comprehensive and precise assessment model for skeletal muscle injury. Its core lies in addressing the inherent heterogeneity, information redundancy, and feature complementarity of multimodal imaging. For the metabolic signatures of PET/CT, mechanical properties of shear wave elastography, and hemodynamic characteristics of DCE-MRI, deep learning enables full-hierarchy fusion spanning the data layer, feature layer, and decision layer: data-layer fusion preserves all original imaging information through registration and pixel-level integration of multimodal images; feature-layer fusion achieves organic integration and complementarity by mapping deep features from distinct modalities to a unified high-dimensional feature space; decision-layer fusion enhances the accuracy and robustness of assessments through weighted integration of diagnostic outcomes from different imaging modalities (108). Compared with traditional multi-source data fusion algorithms, deep learning-based fusion models can automatically learn the weight assignment of features across modalities through end-to-end training, eliminating information redundancy and heterogeneity between modalities. Furthermore, by integrating clinical pathological data and molecular biological test results, a joint deep learning model integrating multimodal imaging, clinical, and molecular data is established to explore the quantitative correlations between imaging features and key molecular events in skeletal muscle injury, enabling reverse deduction from imaging phenotypes to underlying molecular mechanisms and providing non-invasive imaging evidence for mechanistic studies of skeletal muscle injury. In addition, prediction systems based on gradient boosting decision trees (GBDTs), random forests, and deep learning classification and regression models can leverage integrated multi-dimensional features to achieve precise prediction of the occurrence risk, severity, and therapeutic response of immunotherapy-related skeletal muscle injury, offering objective algorithmic decision support for clinical early intervention and treatment regimen optimization.

The AI-assisted multimodal imaging analysis system has completed preliminary algorithm validation and clinical application in clinical research, with its technical effectiveness and practicality supported by data from multiple large-sample studies. Meanwhile, the continuous iteration of deep learning technology has delineated a clear direction for the optimization and clinical translation of this system. Clinical validation data demonstrate that the skeletal muscle injury region segmentation model based on Attention U-Net achieves an accuracy, recall, and F1-score of over 90% in multi-center imaging datasets, with an efficiency dozens of times higher than that of manual segmentation. Furthermore, the intraclass correlation coefficient and Kappa coefficient for injury grading quantification are significantly superior to those of manual assessment, effectively enhancing the standardization and reproducibility of evaluations (109). In terms of efficacy monitoring, the analysis system based on Vision Transformer and multimodal fusion models can dynamically capture changes in imaging features related to skeletal muscle metabolism, elasticity, and vascular microenvironment, enabling the prediction of skeletal muscle injury progression during immunotherapy 1–2 treatment cycles in advance. Its prediction accuracy is 20%-30% higher than that of traditional imaging assessment methods. Currently, this technical system is being continuously optimized by leveraging the latest advances in deep learning technology: the adoption of federated learning addresses the privacy protection challenge in multi-center imaging data sharing, facilitating joint model training with cross-center data; generative adversarial networks and diffusion models can enhance and reconstruct small-sample, low-quality skeletal muscle imaging data, overcoming the limitations of insufficient sample size and uneven data quality in clinical imaging datasets; the design and deployment of lightweight deep learning models (e.g., MobileNet, SqueezeNet) have enabled the implementation of AI imaging analysis models on clinical portable imaging devices (110). In the future, with the in-depth integration of deep learning technology and multimodal imaging technology, the AI-assisted multimodal imaging precise quantitative and integrated analysis system will evolve into a core tool for the diagnosis and management of immunotherapy-related skeletal muscle injury. It will promote the transformation of skeletal muscle injury diagnosis from empirical judgment to precision-, data-, and intelligence-driven practice, providing novel technical support and research insights for the mechanistic investigation and clinical intervention of skeletal muscle injury. The precise assessment system based on multimodal imaging and clinical indicators not only enables the early diagnosis of skeletal muscle injury but also clearly defines the molecular subtypes and severity of injury, thereby offering a key basis for formulating individualized protective strategies and optimal intervention timings.

## 6 Skeletal muscle injury repair and multidimensional protective strategies

### 6.1 Intervention strategies targeting inflammatory cytokines and immune cell infiltration

**Figure 5:**
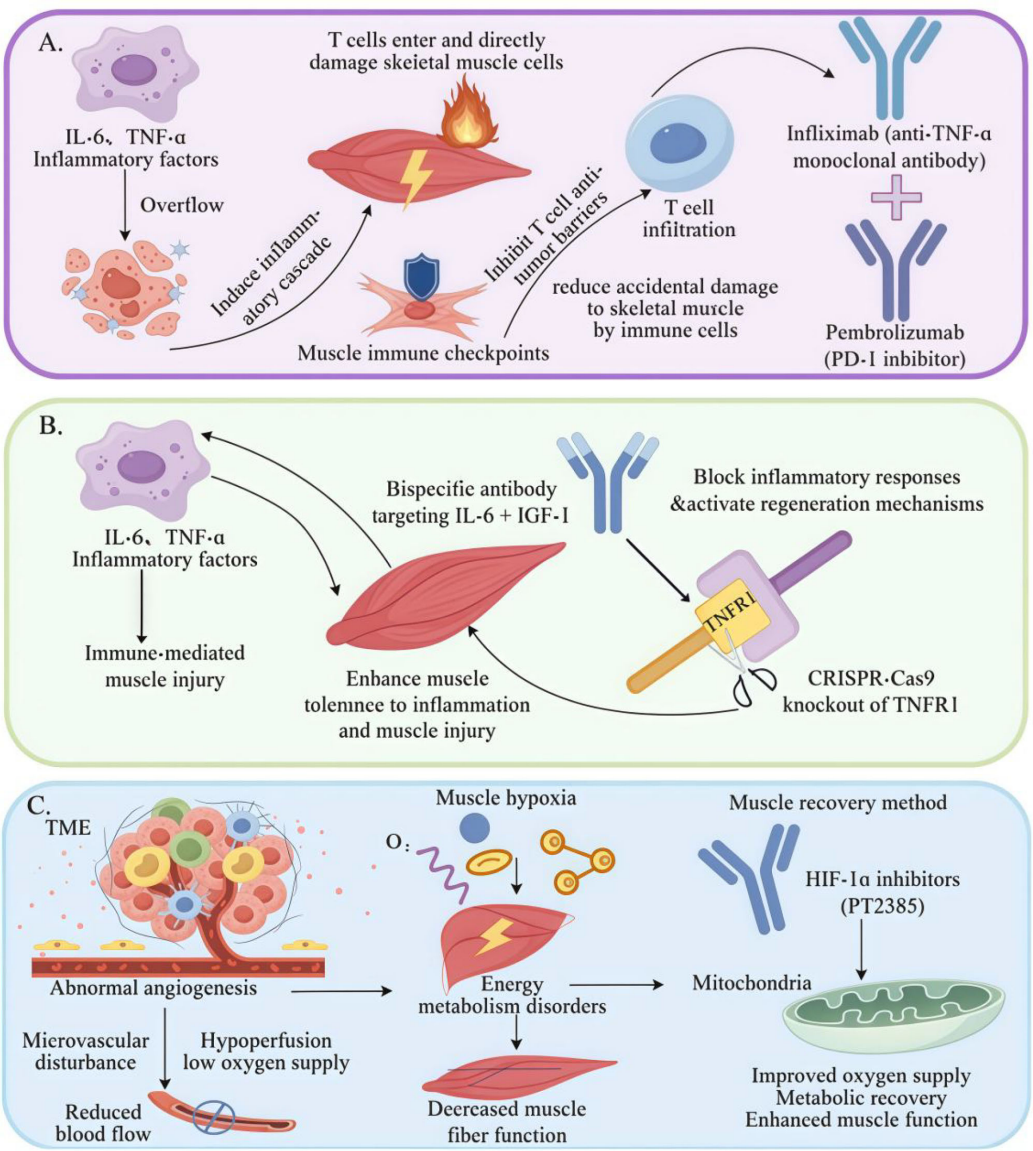
Multidimensional therapeutic strategies for skeletal muscle repair and functional enhancement. **(A)** Immunotherapy-based combination strategy for mitigating skeletal muscle injury. **(B)** Bispecific antibody approach to simultaneously block injury pathways and activate regenerative signaling. **(C)** Skeletal muscle protection strategy based on vascular and metabolic regulation within TME.

Excessive release of inflammatory cytokines and abnormal immune cell infiltration play crucial roles in metabolic dysregulation and structural damage in skeletal muscles. To address these challenges, a combined therapeutic approach involving TNF-α inhibitors and an immune checkpoint blockade has been proposed. By co-administering anti-TNF-α monoclonal antibodies with PD-1 inhibitors, proinflammatory cytokines can be neutralized, while immune checkpoint signaling is disrupted (111). As shown in **Figure 5A**, T cells infiltrated and directly targeted skeletal muscle fibers, while the release of inflammatory cytokines such as TNF-α and IL-6 intensified muscle damage. The combination of anti-TNF-α antibody (Infliximab) and PD-1 inhibitor (Pembrolizumab) effectively reduces cytokine activity and blocks immune checkpoint pathways (112). This combined medication regimen has also been included in the 2025 AANEM Guidelines as a first-line treatment recommendation for moderate-to-severe immunotherapy-related skeletal muscle injury. The guidelines also specify the principles for dosage adjustment and treatment course modification, namely that when the muscle enzyme level returns to within twice the upper limit of normal, the dosage of glucocorticoids can be gradually reduced while maintaining the conventional dosage of TNF-α inhibitors (113). This dual-targeted strategy significantly reduced TNF-α and IL-6 levels in the skeletal muscle, decreased T cell infiltration, and mitigated muscle fiber atrophy and functional impairment. By precisely targeting the underlying pathogenic mechanisms, TNF-α blockade combined with immune checkpoint inhibition enhances vascular perfusion and metabolic homeostasis within the muscle tissue. This approach offers a promising therapeutic model for protecting skeletal muscles during cancer immunotherapy with considerable clinical potential (114). For patients whose symptoms have relieved after treatment with glucocorticoids combined with TNF-α inhibitors, the NCCN Guidelines Version 2025.V1 recommend restarting immunotherapy at a daily dose equivalent to ≤10 mg prednisone, and vedolizumab can be combined at the time of restart to reduce the risk of injury recurrence. For grade 3–4 severe skeletal muscle injury, restarting immunotherapy is still not recommended after treatment, and the original immunotherapy regimen should be permanently discontinued. Meanwhile, the 2025 AANEM Guidelines propose that for refractory skeletal muscle injury unresponsive to glucocorticoid treatment, the complement inhibitor eculizumab can be used for targeted therapy, providing a new direction for the intervention of clinical refractory cases (115).

### 6.2 Bispecific antibody strategies for enhancing skeletal muscle repair

Skeletal muscle injury remains a major challenge for cancer immunotherapy. The release of proinflammatory cytokines and abnormal immune cell infiltration during treatment significantly contribute to muscle damage (116). To address this, bispecific antibodies have emerged as a promising strategy to block immune-mediated damage and activate muscle repair pathways (37). As shown in **Figure 5B, bispecific** antibodies targeting both IL-6 and IGF-1 have been shown to effectively suppressed inflammation while promoting muscle regeneration. Additionally, CRISPR-Cas9 gene editing has been used to knock out TNFR1, thereby enhancing muscle tolerance to inflammation and reducing cytokine-induced injury (117). This combined approach not only mitigates the inflammatory cascade but also significantly promotes muscle repair and regeneration, ultimately enhancing muscle function. Bispecific antibodies, as a form of engineered immunotherapy, provide a dual-action solution by minimizing immune damage and activating regenerative pathways. This innovative strategy holds strong clinical promise for protecting skeletal muscles in cancer immunotherapy (118). The 2025 ESMO Guidelines have put forward monitoring recommendations for the clinical application of bispecific antibodies, specifying that autoantibody levels and muscle enzyme profiles should be monitored monthly during treatment to prevent secondary autoimmune myopathy induced by immune dysregulation. Additionally, the 2025 SITC Consensus Guidelines for the Management of Immune-Related Adverse Events note that the combined administration of bispecific antibodies and myogenic factors can further enhance muscle regeneration efficiency, which is particularly applicable to patients with concurrent myofiber regeneration disorders (119).

### 6.3 Skeletal muscle protection strategies targeting vascular and metabolic regulation within the TME

Abnormal angiogenesis and metabolic dysregulation within the TME are key contributors to skeletal muscle injury during cancer immunotherapy (120). **Figure 5C** highlights this complex pathological process and the associated intervention strategies. Dysregulated angiogenesis within the TME results in dysfunctional microvasculature, leading to inadequate perfusion and hypoxia in muscle tissue. These conditions disrupt energy metabolism and mitochondrial function, and contribute to muscle contractile dysfunction (121). To counteract these effects, the use of HIF-1α inhibitors (e.g., PT2385) has been proposed to improve oxygen delivery and restore metabolic balance. PT2385 enhances hypoxic adaptation mechanisms in muscle cells, increases oxygen availability, revitalizes metabolic activity, and promotes recovery, thereby improving muscle strength and endurance. Animal studies have demonstrated that this strategy significantly reduces muscle injury and facilitates regenerative repair (122). The mechanisms of muscle impact by different cancer treatment modalities are summarized in **Table 1**.

**Table 1:** Mechanisms of muscle impact by different cancer treatment modalities.

Influencing factors	Cytokines	Key molecular pathways	Muscle metabolism	Functional impairment	Clinical manifestations	Related cancers	Reversibility characteristics	Core intervention targets	References
Chemotherapeutic drugs	IL-6, IL-1β, IFN-γ, TNF-α	Ubiquitin-Proteasome System (Atrogin-1, MuRF-1)	Activation of the ubiquitin-proteasome system leading to increased muscle protein breakdown	Decreased muscle strength, muscle atrophy	Muscle weakness, weight loss, fatigue	Lung cancer, Colorectal cancer, Breast cancer	Partial recovery 8–12 weeks after drug withdrawal; irreversible in cachexia-complicated cases	Ubiquitin ligase inhibitors, anabolic drugs	(123, 124)
Immunotherapy	IL-2, IL-6, IFN-γ, TNF-α	NF-κB,JAK/STAT3, Mitochondrial functional pathways	Inflammatory cytokines cause mitochondrial dysfunction, impaired glycolysis and oxidative phosphorylation	Reduced muscle endurance, decreased exercise capacity	Fatigue, muscle pain, reduced exercise tolerance	Melanoma, Non-small cell lung cancer, Renal cell carcinoma	Preliminary recovery 4–8 weeks after withdrawal; partial or complete recovery 12–24 weeks; prolonged to over 6 months in vascular injury-complicated cases	TNF-α/IL-6 neutralizing antibodies, JAK inhibitors, mitochondrial protectants	(125, 126)
Tumor microenvironment & immunotherapy	IL-6, IL-10, IDO, VEGF, TGF-β	HIF-1α,VEGF/VEGFR2, TGF-β/Smad pathways	Altered angiogenesis leading to inadequate muscle oxygen supply, nutritional metabolism disorders	Muscle dysfunction, muscle fibrosis	Muscle stiffness, pain, limited mobility	Liver cancer, Pancreatic cancer, Ovarian cancer	Reversibility depends on vascular reconstruction and metabolic homeostasis recovery; limited recovery in moderate-severe fibrosis cases	Anti-VEGF antibodies, HIF-1α inhibitors, TGF-β receptor antagonists	(127, 128)
Adoptive cell therapy (CAR-T)	CRS-related IL-6, GM-CSF, IFN-γ	JAK/STAT3,NF-κB, Calcineurin pathways	Cytokine storm induces systemic inflammation, direct myotoxicity, and disrupts energy metabolic homeostasis	Severe myasthenia, motor dysfunction	Severe myalgia, fatigue, complicated with myocarditis in some cases	Lymphoma, Multiple myeloma	Reversibility depends on CRS control level; muscle function recovery takes 3–6 months after severe CRS	Anti-IL-6R antibodies, Tocilizumab, immunoadsorption	(129, 130)
Cytokine therapy (IL-2, IFN-α) (IL-2, IFN-α)	High-dose IL-2, IFN-α	JAK/STAT, MAPK/ERK	Directly induce myocyte apoptosis, inhibit muscle satellite cell activation, disrupt energy metabolic homeostasis	Severe muscle wasting, significant decrease in exercise tolerance	Severe fatigue, muscle atrophy	atrophyMelanoma, Renal cell carcinoma	Slow recovery after withdrawal; chronic fatigue syndrome often remains	Anti-cytokine antibodies, metabolic modulators	(131, 132)

Skeletal muscle injury induced by immunotherapy exhibits clear reversibility. Clinical follow-up data demonstrate that most patients achieve initial recovery of skeletal muscle function 4–8 weeks after discontinuing immunotherapy, with partial to complete recovery observed within 12–24 weeks; however, the recovery period may be prolonged to more than 6 months in patients with concurrent severe vascular injury and metabolic disorders (133). The proliferative and differentiative activity of satellite cells, the rate of inflammation resolution, and the degree of recovery of vascular and metabolic homeostasis in the muscle microenvironment are the core factors regulating the recovery process of skeletal muscle injury, as well as critical targets for clinical intervention. Meanwhile, the NCCN Guidelines for Management of Immunotherapy-Related Toxicities (Version 2025.V1) recommends individualized combination medication strategies for skeletal muscle injury mediated by vascular and metabolic disorders, specifying that HIF-1α inhibitors can be co-administered with lactate dehydrogenase inhibitors. By blocking the “tumor-muscle” metabolic axis, this combination regimen inhibits lactate-mediated metabolic reprogramming of skeletal muscle at the source. Additionally, the guidelines mandate that for patients receiving anti-VEGF combined with immunotherapy, high-sensitivity troponin and NT-proBNP should be monitored every cycle during the first 3 treatment cycles to promptly detect early combined muscle-cardiac injury, thereby enabling early screening and intervention (134). Although current preventive and interventional strategies have alleviated immunotherapy-related skeletal muscle injury to a certain extent, these approaches still have inherent limitations, such as insufficient specificity and lack of subtype-targeted interventions. Thus, there is an urgent need to optimize and advance these strategies by integrating the latest mechanistic research findings.

### 6.4 Clinical strategies for perioperative skeletal muscle protection in patients undergoing immunotherapy combined with surgery

The combined application of immunotherapy and surgical resection has emerged as an important model for the comprehensive treatment of malignant tumors. However, their synergistic damaging effects on skeletal muscle metabolism pose new challenges to perioperative management. The systemic inflammatory response induced by surgical stress overlaps with the inflammatory priming state caused by immunotherapy, which can significantly enhance the catabolic burden on skeletal muscle. Moreover, cachexia, as a common baseline condition, leads to insufficient muscle reserve and immunometabolic disorders, further increasing the risk of postoperative complications and potentially compromising the continuity of immunotherapy. Therefore, integrating skeletal muscle protection into the overall perioperative treatment strategy constitutes a critical component for improving the benefits of comprehensive therapy.

Surgical trauma can rapidly activate the neuroendocrine-immune network, triggering the release of glucocorticoids, catecholamines, and inflammatory cytokines such as TNF-α and IL-6. This subsequently activates the NF-κB and JAK/STAT3 signaling pathways, upregulates the expression of myoprotein degradation-related genes (e.g., MuRF1 and Atrogin-1), and induces a “catabolic storm” (135). In patients who have undergone immunotherapy, the inflammatory response is further amplified, which readily surpasses the inflammatory tolerance threshold of skeletal muscle, leading to acute myoprotein loss and reduced muscle strength—an effect that is particularly prominent in the cachectic population.

Cachexia serves as a critical basis for perioperative risk stratification. Patients with this condition are frequently accompanied by mitochondrial dysfunction and suppressed anabolism, which impairs postoperative muscle repair capacity and significantly elevates the risk of infection and delayed wound healing. Meanwhile, the cachexia-associated immunosuppressive state is detrimental to postoperative immune reconstitution and antitumor responses. Therefore, based on cachexia grading and skeletal muscle reserve, priority should be given to optimizing muscle function in moderate-to-severe patients prior to initiating combined treatment regimens (136). Perioperative skeletal muscle protection should be implemented throughout the entire preoperative, intraoperative, and postoperative periods. Preoperatively, resistance exercise combined with nutritional support can be administered for 4–6 weeks to enhance muscle reserve; in patients with high inflammatory levels, inflammatory responses should be cautiously modulated and the timing of immunotherapy should be rationally scheduled. Intraoperatively, anesthesia and medication strategies should be optimized to avoid excessive inflammatory activation. Postoperatively, progressive rehabilitation training should be initiated early, and immunotherapy should be gradually restarted according to the status of muscle recovery to prevent secondary metabolic insult.

In clinical management, a standardized pathway centered on skeletal muscle function assessment should be established, incorporating muscle mass, metabolic status, and inflammatory indicators into routine monitoring. Through multidisciplinary collaboration, synergistic management of immunotherapy, surgery, and skeletal muscle protection can be achieved, thereby reducing perioperative risks, ensuring treatment continuity, and improving long-term patient outcomes.

### 6.5 Clinical strategies to balance efficacy and toxicity in immunotherapy

Against the backdrop of the widespread application of immunotherapy, balancing the maintenance of anti-tumor immune efficacy with the reduction of skeletal muscle dysfunction has emerged as a critical issue for enhancing long-term survival benefits and quality of life. While simple nutritional support can improve energy supply, it fails to intervene in inflammation-driven catabolic pathways at the molecular level. Therefore, comprehensive interventions focusing on inflammatory signal modulation and metabolic function enhancement have gradually become a clinical practice direction. These interventions primarily include targeted drug therapies and functional enhancement strategies centered on resistance exercise, which act on the two key levels of injury control and functional recovery, respectively.

At the pharmacological level, regulating inflammatory and metabolic hub signals is critical for achieving toxicity control without compromising anti-tumor immunity. IL-6 plays a central role in amplifying immune-related inflammation and driving muscle catabolism. IL-6 receptor antagonists (e.g., tocilizumab) alleviate myoprotein degradation by inhibiting the JAK/STAT3 pathway and downregulating the expression of MuRF1 and Atrogin-1 (137). Current evidence indicates that with rational dosing and short-term administration, its impact on immune checkpoint inhibitor efficacy is limited, though individualized assessment remains indispensable. As a key factor linking metabolism and immune tolerance, GDF-15 monoclonal antibody therapy is still in the early stages of research, which initially demonstrates a protective trend for body weight and muscle mass; however, its long-term safety and potential effects on the tumor immune microenvironment remain to be further verified. Therefore, pharmacological interventions should adhere to the principles of “short-term, targeted, and monitorable” with the goal of controlling excessive inflammation while maintaining the threshold of anti-tumor efficacy.

Compared with pharmacological interventions, resistance exercise and preoperative rehabilitation training represent more fundamental and sustainable strategies. Regular resistance training activates the IGF-1/Akt/mTOR axis, promotes myoprotein synthesis, enhances muscle reserve, and improves mitochondrial and oxidative metabolic function, thereby sustaining the activity of CD8^+^ T cells and natural killer cells. Clinical studies have demonstrated that 4–6 weeks of systematic preoperative resistance training increases muscle mass, reduces the risk of perioperative complications, and is not associated with a decrease in the objective response rate of immunotherapy (138).

In clinical practice, stratified management should be implemented based on baseline sarcopenia severity, inflammatory profiles, and treatment regimens. For patients with concurrent cachexia or severe sarcopenia, priority should be given to exercise and nutritional optimization, and short-term targeted anti-inflammatory therapy should be considered in the setting of marked inflammatory elevation. For perioperative patients, emphasis should be placed on the additive effects of postoperative inflammatory peaks on muscle metabolism, with dynamic monitoring of muscle changes and individualized adjustment of rehabilitation progression (139).

Overall, achieving the relative dissociation of efficacy and toxicity does not involve the complete blockage of a single pathway, but rather the reduction of excessive inflammation and metabolic imbalance while maintaining the efficacy of immunotherapy. The combination of molecular targeted modulation and metabolic function enhancement can reduce the risk of skeletal muscle injury to a certain extent, thereby establishing a more sustainable supportive strategy for immunotherapy.

## 7 Limitations and challenges

### 7.1 Methodological and translational gaps

Although significant progress has been made in recent years in elucidating the mechanisms underlying immunotherapy-related skeletal muscle dysfunction, existing mechanistic studies still rely heavily on relatively simplified preclinical models. Most experimental studies employ mouse models with a single tumor type and are conducted under controlled immune activation conditions, making it difficult to truly recapitulate the complex temporal dynamics, cumulative dose effects, and immune exposure scenarios in the context of multimodal combination therapy in clinical cancer treatment. Furthermore, many mechanistic studies tend to focus on a single signaling pathway, while lacking systematic analysis of the hierarchical relationships and networked crosstalk between different pathways. Against the backdrop of the TME-skeletal muscle cross-tissue regulation, a multi-organ immunometabolic process, this relatively “reductionist” research paradigm may oversimplify the actual biological complexity and overlook potential compensatory mechanisms or context-dependent regulatory factors.

At the clinical research level, structural limitations also exist. Most of the available data are derived from single-center retrospective cohort studies, with limited sample sizes and substantial variations in immunotherapy regimens. Systematic stratified analyses remain insufficient with respect to key variables such as tumor classification, immunotherapeutic modalities (e.g., ICIs and ACT), baseline sarcopenia status, metabolic comorbidities, and age stratification. Notably, the representation of elderly patients and high-risk populations receiving combined treatment strategies is significantly inadequate, even though these groups are more prone to developing muscle dysfunction (140). Furthermore, most clinical studies primarily rely on cross-sectional imaging indicators to assess changes in muscle mass, while lacking data support combining longitudinal follow-up and functional measurements. This limitation hinders the dynamic understanding of disease progression, reversibility, and cumulative toxicity.

Insufficient causal inference capacity also constitutes a critical bottleneck in current research. Most existing studies infer potential mechanisms based on correlation analyses among circulating cytokine levels, the degree of immune cell infiltration in skeletal muscle, and changes in imaging-derived muscle parameters. While these associations hold certain biological plausibility, they are inadequate to clarify the direction of causality, nor can they discriminate whether muscle alterations represent a direct toxic effect of immunotherapy or a secondary consequence of systemic metabolic stress (141). Currently, longitudinal studies integrating dynamic immune profiling, serial imaging monitoring, and muscle function assessment remain relatively scarce. Therefore, the temporal sequence between TME remodeling and skeletal muscle dysfunction, as well as the relative contributions of local immune infiltration and systemic inflammatory spillover to pathogenesis, still await further elucidation.

Furthermore, the reproducibility and external validity of research findings also merit attention, particularly in the fields of imaging and multi-omics research. Variations in imaging acquisition protocols, segmentation methodologies, artificial intelligence analysis workflows, and biomarker threshold definitions may all exert substantial impacts on study conclusions. Currently, multi-center validation studies targeting imaging biomarkers or potential protective intervention strategies remain limited, and replication validation in independent cohorts is also relatively scarce. In the absence of methodological standardization and a robust external validation framework, the translation of exploratory research findings into clinically actionable strategies will continue to face practical challenges.

### 7.2 Perioperative and context-specific challenges

As cancer immunotherapy has been progressively integrated into multimodal comprehensive treatment regimens, the interaction between surgical stress and immunotherapy-related skeletal muscle dysfunction remains largely understudied in a systematic manner. Major surgical procedures can trigger a systemic stress response, characterized by acute activation of the hypothalamic-pituitary-adrenal (HPA) axis and the sympathetic nervous system, accompanied by a rapid surge in proinflammatory cytokines such as tumor necrosis factor α (TNF-α) and interleukin 6 (IL-6), as well as the release of various catabolic mediators. In patients with a prior history of ICI or ACT administration, the postoperative cytokine amplification effect may be superimposed on the pre-existing immune activation state, thereby inducing an exaggerated “catabolic storm” (142). However, current studies rarely integrate dynamic perioperative immune changes with skeletal muscle metabolic assessments, resulting in an insufficient understanding of the mechanistic crosstalk between surgical inflammatory responses and muscle vulnerability in the context of immunotherapy.

Cachexia represents another critical yet understudied determinant of perioperative risk. It is characterized by persistent systemic inflammation, accelerated protein catabolism, mitochondrial dysfunction, and suppressed anabolic signaling, alterations that render patients more susceptible to exaggerated catabolic responses after surgical trauma. Although cachexia is well established to correlate with delayed wound healing, elevated infection risk, and prolonged hospital stay, few studies have specifically addressed how pre-existing muscle wasting further modulates perioperative outcomes under immunotherapy regimens. The absence of perioperative risk stratification models incorporating muscle mass, inflammatory biomarkers, and immunotherapy exposure has restricted the precision and individualization of surgical decision-making (143).

Furthermore, no well−defined framework exists for managing exacerbated perioperative muscle catabolism in patients receiving immunotherapy. Prospective studies evaluating the effects of perioperative anti−inflammatory modulation, metabolic support, or optimized immunotherapy timing on postoperative muscle protection remain lacking. Standardized protocols integrating dynamic immune monitoring, nutritional optimization, and systematic muscle function assessment into surgical oncology pathways have not been established. Addressing these research gaps is critical to mitigating the escalation of postoperative complication risk, improving recovery trajectories, and enhancing long−term prognosis, while also providing a novel theoretical foundation for surgical management strategies in the immunotherapy era.

## 8 Conclusions and future perspectives

This review systematically integrates current advances in cancer immunotherapy-induced skeletal muscle dysfunction and establishes a multi-level analytical framework spanning mechanistic studies, clinical observations, multimodal imaging techniques, and emerging intervention strategies. A consensus is emerging from available evidence: immunotherapy-related skeletal muscle dysfunction is not an isolated adverse event, but a systemic biological manifestation of peripheral immune regulation, representing a complex, multifactorial, and cross-organ crosstalk pathological process. Within this framework, inflammatory cytokine spillover, aberrant immune cell infiltration, metabolic reprogramming, and TME-associated vascular dysfunction interact interdependently and collectively constitute the core basis for skeletal muscle vulnerability.

Numerous basic and clinical studies consistently indicate that the sustained activation of the NF-κB and JAK/STAT3 signaling pathways constitutes a critical molecular hub linking systemic immune activation to myoprotein degradation, regeneration inhibition, and catabolic amplification (144). Meanwhile, the value of multimodal imaging techniques (e.g., PET/CT, shear wave elastography, and DCE-MRI) in the noninvasive assessment of skeletal muscle metabolic abnormalities, mechanical changes, and vascular microenvironment disorders has been repeatedly validated. These converging lines of evidence collectively establish a relatively robust mechanistic model, wherein sustained inflammatory signal activation and metabolic-vascular imbalance form the core driver axis of skeletal muscle dysfunction in the context of immunotherapy.

Nevertheless, substantial inconsistencies remain among current studies regarding the dominant mechanisms and their relative contributions to the pathogenic cascade. While some investigations highlight dysregulated Tregs and aberrant local immune cell infiltration as decisive factors, others posit that synergistic cytokine amplification and systemic metabolic stress constitute the primary driving forces (145). Such discrepancies largely stem from methodological heterogeneity, including variations in tumor types, immunotherapeutic modalities (ICIs, ACT, or cytokine therapy), inconsistencies between animal models and clinical specimens, as well as differences in skeletal muscle injury criteria and follow-up intervals. Furthermore, cross-sectional study designs limit the dynamic understanding of injury reversibility, cumulative toxicity, and long-term functional outcomes.

From the perspective of evidence maturity, distinct stratification also exists across different research directions. The cytokine−mediated activation of the ubiquitin-proteasome system, the central role of the NF−κB/JAK/STAT3 axis in myoprotein degradation, and the potential protective effects of anti−inflammatory interventions have established a robust experimental and translational evidence base. In contrast, noncoding RNA−mediated cross−tissue crosstalk between tumors and skeletal muscle, artificial intelligence−assisted multimodal imaging integration, bispecific antibody strategies, and the application of gene−editing techniques in protecting against muscle injury remain at the exploratory stage, largely supported by small−sample or preliminary experimental data (146). Moreover, within the surgical and perioperative context, dynamic TME-skeletal muscle crosstalk may further amplify postoperative catabolic responses and impair recovery trajectories and long−term outcomes, yet the corresponding mechanisms and risk−stratification models remain to be established. Elderly patients, individuals with pre−existing sarcopenia, and high−risk populations undergoing multimodal therapy are underrepresented in current studies, limiting the external generalizability of the conclusions.

Future research is urgently required to shift from single association−based description toward an integrated, longitudinal, and multilevel research paradigm. Establishing prospective cohorts incorporating dynamic immune profiling, quantitative multimodal imaging assessment, muscle function measurement, and multi−omics integration will help clarify the causal hierarchy and key driving mechanisms. Mechanistically, studies should expand from single−pathway investigations to network−level exploration of TME–skeletal muscle cross−tissue crosstalk, to identify intervention targets with greater translational potential. Meanwhile, standardization and multi−center validation of imaging acquisition protocols and artificial intelligence analysis pipelines should be promoted to improve the reproducibility and external validity of findings (147).

At the practical implementation level, future research can be advanced in four sequential stages: mechanistic research, biomarker identification, diagnostic integration, and clinical intervention. For mechanistic research, cutting-edge technologies including single-cell RNA sequencing, spatial transcriptomics, and immunometabolomics should be integrated to systematically dissect the immune and metabolic crosstalk networks between the tumor microenvironment and skeletal muscle, with a key focus on identifying the critical cell subsets and signaling molecules that drive skeletal muscle injury. In terms of biomarker development, multidimensional indicators such as circulating inflammatory cytokine profiles, muscle-derived microRNAs, exosomal molecules, and radiomic features can be further explored to establish a comprehensive biomarker system applicable for early risk prediction and dynamic monitoring. For diagnostic integration, the combined application of multimodal imaging techniques (e.g., PET/CT, MRI, and shear wave elastography) with immune- and metabolic-related biomarkers should be promoted, and artificial intelligence approaches leveraged to integrate imaging and molecular data, thereby constructing predictive models for the early identification of immunotherapy-associated muscle toxicity. Additionally, future prospective multicenter cohort studies should enroll high-risk populations such as elderly patients and those with pre-existing sarcopenia to establish a more precise risk stratification system. In translational and interventional research, future efforts should prioritize the exploration of comprehensive intervention strategies, including anti-inflammatory modulation, metabolic regulation, nutritional support, and individualized exercise rehabilitation protocols. Simultaneously, randomized controlled trials can be conducted within the framework of perioperative management to evaluate the potential of these interventions to reduce skeletal muscle toxicity while preserving anti-tumor efficacy of immunotherapy.

Ultimately, translational research integrating perioperative management, metabolic modulation, and targeted anti−inflammatory strategies is expected to achieve the relative dissociation between antitumor efficacy and skeletal muscle toxicity. This will advance risk stratification and individualized management within a precision medicine framework, thereby providing systematic theoretical support and practical routes for optimizing comprehensive cancer care in the immunotherapy era.

## References

[1] Baghban R Zendehdel N Rajabibazl M Mortezaee K Salehi R Ghayour-Mobarhan M . Tumor microenvironment complexity and therapeutic implications at a glance. Cell Commun Signaling. (2020) 18:59. doi: 10.1186/s12964-020-0530-4 32264958 PMC7140346

[2] Biray Avci C Göktaş Ç Demircan B Çelik Ö Akkoç Y Erdem B . Tumor microenvironment and cancer metastasis: molecular mechanisms and therapeutic implications. Front Pharmacol. (2024) 15:1442888. doi: 10.3389/fphar.2024.1442888 39600368 PMC11588459

[3] Ma S Wang Y Zhang Y Li Y Liu X Zhao H . Unraveling the triad of immunotherapy, tumor microenvironment, and skeletal muscle biomechanics in oncology. Front Immunol. (2025) 16:1572821. doi: 10.3389/fimmu.2025.1572821 40242775 PMC12000078

[4] Yang J Zhang L Li H Wang Y Liu S Chen X . Tumor secretome shapes the immune landscape during cancer progression. J Exp Clin Cancer Res. (2025) 44:47. doi: 10.1186/s13046-025-03302-0 39930476 PMC11809007

[5] Andreev G Petrov I Ivanova A Kiselev A Smirnova V Zolotarenko E . Spatial correlation of the extracellular matrix to immune cell phenotypes in the tumor boundary of clear cell renal cell carcinoma revealed by cyclic immunohistochemistry. Lab Invest. (2025) 105:104130. doi: 10.1016/j.labinv.2025.104130 40120686

[6] Zhao Y Zhang L Wang X Li J Liu Y Chen H . Stromal cells in the tumor microenvironment: accomplices of tumor progression? Cell Death Dis. (2023) 14:587. doi: 10.1038/s41419-023-06110-6 37666813 PMC10477351

[7] Mao X Xu J Wang W Li J Zhang Y Liu Z . Crosstalk between cancer-associated fibroblasts and immune cells in the tumor microenvironment: new findings and future perspectives. Mol Cancer. (2021) 20:131. doi: 10.1186/s12943-021-01428-1 34635121 PMC8504100

[8] He S Zheng L Qi C . Myeloid-derived suppressor cells (MDSCs) in the tumor microenvironment and their targeting in cancer therapy. Mol Cancer. (2025) 24:5. doi: 10.1186/s12943-024-02208-3 39780248 PMC11707952

[9] Kelly PN . The cancer immunotherapy revolution. Science. (2018) 359:1344–5. doi: 10.1126/science.359.6382.1344 29567702

[10] Ribas A Wolchok JD . Cancer immunotherapy using checkpoint blockade. Science. (2018) 359:1350–5. doi: 10.1126/science.aar4060 29567705 PMC7391259

[11] Sun W Hu S Wang X . Advances and clinical applications of immune checkpoint inhibitors in hematological Malignancies. Cancer Commun. (2024) 44:1071–97. doi: 10.1002/cac2.12564 39073258 PMC11492363

[12] VanderVeen BN Murphy EA Carson JA . The impact of immune cells on the skeletal muscle microenvironment during cancer cachexia. Front Physiol. (2020) 11:1037. doi: 10.3389/fphys.2020.01037 32982782 PMC7489038

[13] Zhang ML Jin WL . Dysregulated energy homeostasis at the crossroads of cancer–depression comorbidity: a brain–body–tumor interaction perspective. Cancer Lett. (2026) 645:218351. doi: 10.1016/j.canlet.2026.218351 41748012

[14] Berriel Diaz M Rohm M Herzig S . Cancer cachexia: multilevel metabolic dysfunction. Nat Metab. (2024) 6:2222–45. doi: 10.1038/s42255-024-01167-9 39578650

[15] Ma S Wang Y Zhang Y Li Y Liu X Zhao H . Tumor microenvironment and immune-related myositis: addressing muscle wasting in cancer immunotherapy. Front Immunol. (2025) 16:1580108. doi: 10.3389/fimmu.2025.1580108 40386783 PMC12081358

[16] de Visser KE Joyce JA . The evolving tumor microenvironment: from cancer initiation to metastatic outgrowth. Cancer Cell. (2023) 41:374–403. doi: 10.1016/j.ccell.2023.02.004 36917948

[17] Zhang M Li J Wang Y Liu S Zhang L Chen X . Advances in cancer immunotherapy: historical perspectives, current developments, and future directions. Mol Cancer. (2025) 24:136. doi: 10.1186/s12943-025-02305-x 40336045 PMC12057291

[18] Colardelle Y Smith J Jones A Brown K Garcia M Martinez R . Cancer cachexia prevalence is underestimated in medical records of patients in a regional tertiary hospital. J Cachexia Sarcopenia Muscle. (2026) 17:e70225. doi: 10.1002/jcsm.13245 41704163 PMC12914221

[19] Sun D Wang L Zhang Y Li H Liu X Zhao J . Cancer burden and trends in China: a review and comparison with Japan and South Korea. Chin J Cancer Res. (2020) 32:129–39. doi: 10.21147/j.issn.1000-9604.2020.02.02 32410791 PMC7219092

[20] Ferrara M Rossi F Bianchi M Russo A Ferrari S Romano G . Cancer cachexia as a multiorgan failure: reconstruction of the crime scene. Front Cell Dev Biol. (2022) 10:960341. doi: 10.3389/fcell.2022.960341 36158184 PMC9493094

[21] Xue D Han Y Wang Z Liu C Zhang Q Li N . Sarcopenia predicts immune-related adverse events due to anti-PD-1/PD-L1 therapy in patients with advanced lung cancer. Front Oncol. (2024) 14:1450020. doi: 10.3389/fonc.2024.1450020 39376979 PMC11456396

[22] Mauriello A Esposito F Conte S D'Auria F De Rosa M Fabbricini D . Immune checkpoint inhibitor-associated myocarditis: risk, diagnosis, and clinical impact. J Clin Med. (2026) 15:345. doi: 10.3390/jcm15020814 41598751 PMC12841761

[23] Tufail M Jiang CH Li N . Altered metabolism in cancer: insights into energy pathways and therapeutic targets. Mol Cancer. (2024) 23:203. doi: 10.1186/s12943-024-02119-3 39294640 PMC11409553

[24] Erasha AM Alghamdi AS Alshahrani S Alzahrani AJ Alghamdi B Alghamdi M . The role of the tumor microenvironment (TME) in advancing cancer therapies: immune system interactions, tumor-infiltrating lymphocytes (TILs), and the role of exosomes and inflammasomes. Int J Mol Sci. (2025) 26:1892. doi: 10.3390/ijms26062716 40141358 PMC11942452

[25] Petitprez F Levy S Chenache H Amara K Mechta-Grigoriou F Imbaud S . B cells are associated with survival and immunotherapy response in sarcoma. Nature. (2020) 577:556–60. doi: 10.1038/s41586-019-1906-8 31942077

[26] Li T Zhang Y Wang Y Liu X Zhao H Li J . Research progress and future perspectives of prodrug strategies for immune checkpoint inhibitors in cancer immunotherapy. Crit Rev Oncol/Hematol. (2025) 214:104905. doi: 10.1016/j.critrevonc.2025.104905 40834987

[27] Liu J Chen X Zhang L Wang Z Li H Liu S . Reprogramming the immunosuppressive tumor microenvironment through nanomedicine: an immunometabolism perspective. eBioMedicine. (2024) 107:105301. doi: 10.1016/j.ebiom.2024.105301 39178747 PMC11388279

[28] Meng L Zhang Y Li J Wang Y Liu X Zhao H . Mechanisms of immune checkpoint inhibitors: insights into the regulation of circular RNAs involved in cancer hallmarks. Cell Death Dis. (2024) 15:3. doi: 10.1038/s41419-023-06389-5 38177102 PMC10766988

[29] Cui JW Zhang L Wang Y Li J Liu X Zhao H . Tumor immunotherapy resistance: revealing the mechanism of PD-1/PD-L1-mediated tumor immune escape. Biomed Pharmacotherapy. (2024) 171:116203. doi: 10.1016/j.biopha.2024.116203 38280330

[30] Kennedy PT Smith A Jones B Brown C Davis D Evans E . Soluble CTLA-4 attenuates T cell activation and modulates anti-tumor immunity. Mol Ther. (2024) 32:457–68. doi: 10.1016/j.ymthe.2023.11.015 38053333 PMC10861965

[31] Liu Y Zhang Y Wang Y Li J Liu X Zhao H . Concurrent immune checkpoint blockade for enhanced cancer immunotherapy utilizing engineered hybrid nanovesicles. Front Pharmacol. (2024) 15:1442888. doi: 10.3389/fphar.2024.1442888 39588148 PMC11586202

[32] Lin X Zhang L Wang Y Li J Liu X Zhao H . Regulatory mechanisms of PD-1/PD-L1 in cancers. Mol Cancer. (2024) 23:108. doi: 10.1186/s12943-024-02023-w 38762484 PMC11102195

[33] Zhao Y Zhang L Wang X Li J Liu Y Chen H . Tumor infiltrating lymphocyte (TIL) therapy for solid tumor treatment: progressions and challenges. Cancers (Basel). (2022) 14:4219. doi: 10.3390/cancers14174219 36077696 PMC9455018

[34] Ma S Wang Y Zhang Y Li Y Liu X Zhao H . Concentration optimization of combinatorial drugs using Markov chain-based models. BMC Bioinf. (2021) 22:451. doi: 10.1186/s12859-021-04364-5 34548014 PMC8456646

[35] Qiu M Li P . CRISPR/Cas-based diagnostics and gene therapy. Bio Integ. (2021) 2:121–9. doi: 10.15212/bioi-2020-0048

[36] Zhou Y Wang Y Zhang Y Li J Liu X Zhao H . Therapeutic potential of tumor-associated neutrophils: dual role and phenotypic plasticity. Signal Transduc Targeted Ther. (2025) 10:178. doi: 10.1038/s41392-025-02242-7 40461514 PMC12134342

[37] Niu J Wang Y Zhang Y Li J Liu X Zhao H . Novel CAR-T cells specifically targeting Nectin4 exhibit effective anti-tumor efficacy in bladder cancer cell lines. Bio Integ. (2025) 6:1–11. doi: 10.15212/bioi-2025-0041

[38] Liu R Li H-F Li S . PD-1-mediated inhibition of T cell activation: Mechanisms and strategies for cancer combination immunotherapy. Cell Insight. (2024) 3:100146. doi: 10.1016/j.cellin.2024.100146 38425643 PMC10901852

[39] Pal SK Plimack ER Atkins MB McDermott DF Heng DY Bearss DJ . CD70-targeted allogeneic CAR T-cell therapy for advanced clear cell renal cell carcinoma. Cancer Discov. (2024) 14:1176–89. doi: 10.1158/2159-8290.CD-24-0102 38583184 PMC11215406

[40] Lin G Wang Y Zhang Y Li J Liu X Zhao H . *In vivo* iron-based coordination assembly for disease diagnosis and treatment. Bio Integ. (2023) 4:70–2. doi: 10.15212/bioi-2022-0016

[41] Manzano S Caffarel MM . Cytokine-centered strategies to boost cancer immunotherapy. Mol Oncol. (2025) 19:579–83. doi: 10.1002/1878-0261.13512 39918251 PMC11887662

[42] Datta N . Stem cell therapy for SARS-CoV-2 and influenza virus infections. Bio Integ. (2024) 5:1–13. doi: 10.15212/bioi-2024-0016

[43] Jung K Lee S Park J Kim S Choi J Lee J . Improved intratumoral penetration of IL12 immunocytokine enhances the antitumor efficacy. Front Immunol. (2022) 13:1034774. doi: 10.3389/fimmu.2022.1034774 36405748 PMC9667294

[44] Li J Wang Y Zhang Y Liu X Zhao H Chen X . Vitcylation of lysine: the novel mechanism of vitamin C in tumor treatment. Bio Integ. (2025) 6:1–6. doi: 10.15212/bioi-2025-0057

[45] Shouse AN Villarino AV Malek TR . Interleukin-2 receptor signaling acts as a checkpoint that influences the distribution of regulatory T cell subsets. iScience. (2024) 27:111248. doi: 10.1016/j.isci.2024.111248 39759017 PMC11700635

[46] Yang J Zhang Y Wang Y Li J Liu X Zhao H . Plant extracellular vesicles: a promising bionic medicine platform for disease treatment and drug delivery. Interdiscip Med. (2025) 3:e20240117. doi: 10.1002/idm2.12017 41837764

[47] Lin Y Zhang Y Wang Y Li J Liu X Zhao H . New insights on anti-tumor immunity of CD8(+) T cells: cancer stem cells, tumor immune microenvironment and immunotherapy. J Transl Med. (2025) 23:341. doi: 10.1186/s12967-025-06291-y 40097979 PMC11912710

[48] Raskov H Petersen HL Hokland M Brünner N Svane IM Andersen MH . Natural killer cells in cancer and cancer immunotherapy. Cancer Lett. (2021) 520:233–42. doi: 10.1016/j.canlet.2021.09.025 34302920

[49] Zhang X Li J Wang Y Zhang Y Liu X Zhao H . The cell cycle regulator p16 promotes tumor infiltrated CD8(+) T cell exhaustion and apoptosis. Cell Death Dis. (2024) 15:339. doi: 10.1038/s41419-024-06721-7 38750022 PMC11096187

[50] Nirschl CJ Magiera LC Rojas JL Spranger S Scherer DC Gnjatic S . mWTX-330, an IL-12 INDUKINE molecule, activates and reshapes tumor-infiltrating CD8+ T and NK cells to generate antitumor immunity. Cancer Immunol Res. (2023) 11:962–77. doi: 10.1158/2326-6066.CIR-22-0705 37074216 PMC10320472

[51] Zhang A Li J Wang Y Zhang Y Liu X Zhao H . Regulatory T cells in immune checkpoint blockade antitumor therapy. Mol Cancer. (2024) 23:251. doi: 10.1186/s12943-024-02156-y 39516941 PMC11545879

[52] Yang B Li J Wang Y Zhang Y Liu X Zhao H . An Asia-specific variant of human IgG1 represses colorectal tumorigenesis by shaping the tumor microenvironment. J Clin Invest. (2022) 132:e154678. doi: 10.1172/JCI153454 35133976 PMC8920342

[53] Lan X Li J Wang Y Zhang Y Liu X Zhao H . Positron emission tomography imaging sheds new light on hypoxia and antitumor therapies. Interdiscip Med. (2023) 1:e20230025. doi: 10.1002/INMD.20230002 41837764

[54] Fang J Li J Wang Y Zhang Y Liu X Zhao H . Exploring the crosstalk between endothelial cells, immune cells, and immune checkpoints in the tumor microenvironment: new insights and therapeutic implications. Cell Death Dis. (2023) 14:586. doi: 10.1038/s41419-023-06119-x 37666809 PMC10477350

[55] Pryce BR Singh M Aumann NK Wang J Wijesuriya SS Clements K . Muscle inflammation is regulated by NF-κB from multiple cells to control distinct states of wasting in cancer cachexia. Cell Rep. (2024) 43:114925. doi: 10.1016/j.celrep.2024.114925 39475511 PMC11774514

[56] Nelke C Li J Wang Y Zhang Y Liu X Zhao H . K(2P)2.1 is a regulator of inflammatory cell responses in idiopathic inflammatory myopathies. J Autoimmun. (2024) 142:103136. doi: 10.1016/j.jaut.2023.103136 37935063

[57] Wang C Li J Wang Y Zhang Y Liu X Zhao H . Immune function assessing of TIM3/CD28-modified CD19 CAR-T cells and general CD19 CAR-T cells through a high-throughput single-cell microarray platform. Interdiscip Med. (2024) 2:e20230030. doi: 10.1002/idm2.12030 41837764

[58] Luo Y Li J Wang Y Zhang Y Liu X Zhao H . Solid cancer-directed CAR T cell therapy that attacks both tumor and immunosuppressive cells via targeting PD-L1. Mol Ther Oncol. (2024) 32:200891. doi: 10.1016/j.omton.2024.200891 39498357 PMC11532918

[59] Lee CS Kim JH Park JH Lee JH Kim YS Park SJ . IKK-mediated TRAF6 and RIPK1 interaction stifles cell death complex assembly leading to the suppression of TNF-α-induced cell death. Cell Death Dif. (2023) 30:1575–84. doi: 10.1038/s41418-023-01161-w 37085671 PMC10244383

[60] Glover HL Li J Wang Y Zhang Y Liu X Zhao H . Mitochondria and cell death. Nat Cell Biol. (2024) 26:1434–46. doi: 10.1038/s41556-024-01429-4 38902422

[61] Li H Li J Wang Y Zhang Y Liu X Zhao H . Cell death: the underlying mechanisms of photodynamic therapy for skin diseases. Interdiscip Med. (2025) 3:e20240057. doi: 10.1002/idm2.12057 41837764

[62] Kamiya M Tajima Y Imai Y Ito N Nakamura T Suzuki K . Targeting necroptosis in muscle fibers ameliorates inflammatory myopathies. Nat Commun. (2022) 13:166. doi: 10.1038/s41467-021-27875-4 35013338 PMC8748624

[63] Yang B Li J Wang Y Zhang Y Liu X Zhao H . Applications of bioactive herbal extracts in dressing materials for skin wound repair: ingredients, mechanisms and innovations. Interdiscip Med. (2024) 3:e20240117. doi: 10.1002/idm2.12017 41837764

[64] Pelosi L Reinders C van der Velden S van der Meulen J van der Laarse WJ van der Velden J . Sustained systemic levels of IL-6 impinge early muscle growth and induce muscle atrophy and wasting in adulthood. Cells. (2021) 10:1723. doi: 10.3390/cells10071723 34359985 PMC8306542

[65] Zöphel S Li J Wang Y Zhang Y Liu X Zhao H . Identification of molecular candidates which regulate calcium-dependent CD8(+) T-cell cytotoxicity. Mol Immunol. (2023) 157:202–13. doi: 10.1016/j.molimm.2023.04.009 37075611

[66] Li AQ Fang JH . Anti-angiogenic therapy enhances cancer immunotherapy: mechanism and clinical application. Interdiscip Med. (2024) 2:e20230025. doi: 10.1002/idm2.12025 41837764

[67] Rana PS Chakraborty A Dutta S Das S Gupta A Singh R . Immunoproteasome activation expands the MHC class I immunopeptidome, unmasks neoantigens, and enhances T-cell anti-myeloma activity. Mol Cancer Ther. (2024) 23:1743–60. doi: 10.1158/1535-7163.MCT-23-0931 39210605 PMC11612626

[68] Guo Q Li Y Wang X Zhang L Liu J Chen Y . NF-κB in biology and targeted therapy: new insights and translational implications. Signal Transduc Targeted Ther. (2024) 9:53. doi: 10.1038/s41392-024-01757-9 38433280 PMC10910037

[69] Wang T Zhou D Hong Z . Sarcopenia and cachexia: molecular mechanisms and therapeutic interventions. MedComm. (2025) 6:e70030. doi: 10.1002/mco2.70030 39764565 PMC11702502

[70] Lorenc P Komosińska-Kaszubska E Władysław A Musielak Z Klinghammer K Jankowska-Stepien E . Physiological and tumor-associated angiogenesis: key factors and therapy targeting VEGF/VEGFR pathway. Biomed Pharmacotherapy. (2024) 180:117585. doi: 10.1016/j.biopha.2024.117585 39442237

[71] Manning D Rivera EJ Santana LF . The life cycle of a capillary: mechanisms of angiogenesis and rarefaction in microvascular physiology and pathologies. Vascul Pharmacol. (2024) 156:107393. doi: 10.1016/j.vph.2024.107393 38857638 PMC12051481

[72] Mao Y Li J Wang Y Zhang Y Liu X Zhao H . Hypoxia induces mitochondrial protein lactylation to limit oxidative phosphorylation. Cell Res. (2024) 34:13–30. doi: 10.1038/s41422-023-00864-6 38163844 PMC10770133

[73] Ye Y Li J Wang Y Zhang Y Liu X Zhao H . *In vitro* study: HIF-1α-dependent glycolysis enhances NETosis in hypoxic conditions. Front Immunol. (2025) 16:1583587. doi: 10.3389/fimmu.2025.1583587 40356921 PMC12066692

[74] Hu M Li J Wang Y Zhang Y Liu X Zhao H . The regulatory role of autophagy between TAMs and tumor cells. Cell Biochem Funct. (2024) 42:e3984. doi: 10.1002/cbf.3984 38494666

[75] Yan Y Li J Wang Y Zhang Y Liu X Zhao H . MiR-126-5p derived from bone marrow mesenchymal stem cell exosomes promotes skeletal muscle regeneration by regulating FBXO32/MyoD signaling. Acta Physiol Oxf. (2025) 241:e70114. doi: 10.1111/apha.70114 41025492

[76] Matsubara S Kowa H Akiyama H Ito N Imai Y Suzuki K . Tertiary lymphoid organs in the inflammatory myopathy associated with PD-1 inhibitors. J Immunother Cancer. (2019) 7:256. doi: 10.1136/jitc-2019-000648 31533865 PMC6751882

[77] Wang Y Li J Zhang Y Liu X Zhao H Chen X . PDP1 promotes the progression of breast cancer through STAT3 pathway. Cell Biochem Funct. (2024) 42:e3994. doi: 10.1002/cbf.3994 38566355

[78] Pang QM Li J Wang Y Zhang Y Liu X Zhao H . Peripheral blood-derived mesenchymal stem cells modulate macrophage plasticity through the IL-10/STAT3 pathway. Stem Cells Int. (2022) 2022:5181241. doi: 10.1155/2022/5181241 35450344 PMC9017453

[79] Nazari-Khanamiri F Li J Wang Y Zhang Y Liu X Zhao H . Tumor cells-derived exosomal noncoding RNAs in cancer angiogenesis: molecular mechanisms and prospective. Cell Biochem Funct. (2023) 41:1008–15. doi: 10.1002/cbf.1008 37843018

[80] Liu T Li J Wang Y Zhang Y Liu X Zhao H . Role of human monocarboxylate transporter 1 (hMCT1) and 4 (hMCT4) in tumor cells and the tumor microenvironment. Cancer Manag Res. (2023) 15:957–75. doi: 10.2147/CMAR.S421771 37693221 PMC10487743

[81] Li X Li J Wang Y Zhang Y Liu X Zhao H . Regulation of calcium homeostasis in endoplasmic reticulum-mitochondria crosstalk: implications for skeletal muscle atrophy. Cell Commun Signal. (2025) 23:17. doi: 10.1186/s12964-024-02014-w 39789595 PMC11721261

[82] Zhang L Li J Wang Y Zhang Y Liu X Zhao H . Lactate transported by MCT1 plays an active role in promoting mitochondrial biogenesis and enhancing TCA flux in skeletal muscle. Sci Adv. (2024) 10:eadn4508. doi: 10.1126/sciadv.adn4508 38924407 PMC11204292

[83] Wang F Li J Wang Y Zhang Y Liu X Zhao H . Canonical Wnt signaling promotes HSC glycolysis and liver fibrosis through an LDH-A/HIF-1α transcriptional complex. Hepatology. (2024) 79:606–23. doi: 10.1097/HEP.0000000000000569 37733267 PMC10871634

[84] Schwiete C Li J Wang Y Zhang Y Liu X Zhao H . Overlaps of skeletal muscle fatigue and skeletal muscle damage: the muscle injury continuum. Sports Med - Open. (2025) 11:73. doi: 10.1186/s40798-025-00876-z 40495076 PMC12151979

[85] Park HN Li J Wang Y Zhang Y Liu X Zhao H . LRG1 promotes ECM integrity by activating the TGF-β signaling pathway in fibroblasts. Int J Mol Sci. (2023) 24:12456. doi: 10.3390/ijms241512456 37569820 PMC10418909

[86] Arnholdt C Li J Wang Y Zhang Y Liu X Zhao H . Depletion of γδ T cells leads to reduced angiogenesis and increased infiltration of inflammatory M1-like macrophages in ischemic muscle tissue. Cells. (2022) 11:1487. doi: 10.1186/s40425-019-0736-4 35563796 PMC9102774

[87] Absalón-Aguilar A Li J Wang Y Zhang Y Liu X Zhao H . TRIM63 and Atrogin-1 are key drivers of systemic and muscle inflammation in patients with idiopathic inflammatory myopathies. Clin Exp Rheumatol. (2025) 43:326–33. doi: 10.55563/clinexprheumatol/p2lma6 39946176

[88] Ashrafizadeh M Li J Wang Y Zhang Y Liu X Zhao H . Non-coding RNA-based regulation of inflammation. Semin Immunol. (2022) 59:101606. doi: 10.1016/j.smim.2022.101606 35691882

[89] Wagner J Li J Wang Y Zhang Y Liu X Zhao H . TRAF2 and RIPK1 redundantly mediate classical NFκB signaling by TNFR1 and CD95-type death receptors. Cell Death Dis. (2025) 16:35. doi: 10.1038/s41419-024-07325-x 39837830 PMC11751453

[90] Huang B Lang X Li X . The role of IL-6/JAK2/STAT3 signaling pathway in cancers. Front Oncol. (2022) 12:1023177. doi: 10.3389/fonc.2022.1023177, PMID: 36591515 PMC9800921

[91] Roberson PA Jefferson LS Kimball SR . Convergence of signaling pathways in mediating actions of leucine and IGF-1 on mTORC1 in L6 myoblasts. Am J Physiol Cell Physiol. (2022) 323:C804–12. doi: 10.1152/ajpcell.00183.2022 35912992 PMC9448342

[92] Subhan I Alam S Hossain I Islam M Rahman M Akter S . Toxicity and genotoxicity of AlCl3 in transgenic Drosophila melanogaster (hsp-70 lacZ) Bg9. Mutat Res Genet Toxicol Environ Mutagen. (2026) 909:503905. doi: 10.1016/j.mrgentox.2025.503905 41519509

[93] Mun Y Li J Wang Y Zhang Y Liu X Zhao H . cGAS–STING–NF-κB axis mediates rotenone-induced NLRP3 inflammasome activation through mitochondrial DNA release. Antioxidants. (2025) 14:1276. doi: 10.3390/antiox14111276 41300433 PMC12649239

[94] Zhao T Li J Wang Y Zhang Y Liu X Zhao H . Prognostic impact of sarcopenia on 5-year overall and progression-free survival in lung cancer patients: a prospective cohort study. Front Nutr. (2026) 13:1727652. doi: 10.3389/fnut.2026.1727652 41717028 PMC12913170

[95] Nishidate A Li J Wang Y Zhang Y Liu X Zhao H . Human PBMC-based humanized mice exhibit myositis features and serve as a drug evaluation model. Inflammation Regener. (2025) 45:1. doi: 10.1186/s41232-025-00365-6 39810259 PMC11734410

[96] Pantel AR Li J Wang Y Zhang Y Liu X Zhao H . PET imaging of metabolism, perfusion, and hypoxia: FDG and beyond. Cancer J. (2024) 30:159–69. doi: 10.1097/PPO.0000000000000602 38753750 PMC11101148

[97] Chao PY Li J Wang Y Zhang Y Liu X Zhao H . Shear wave elasticity measurements of three-dimensional cancer cell cultures using laser speckle contrast imaging. Sci Rep. (2018) 8:14470. doi: 10.1038/s41598-018-32763-x 30262836 PMC6160414

[98] Saucedo AM Li J Wang Y Zhang Y Liu X Zhao H . Multimodal imaging of the tumor microenvironment and biological responses to immune therapy. BioMed Microdev. (2018) 20:105. doi: 10.1007/s10544-018-0347-8 30535532 PMC7466906

[99] Gao Y Li J Wang Y Zhang Y Liu X Zhao H . Metabolic characterization and radiomics-based composite model for breast cancer immune microenvironment types using (18)F-FDG PET/CT. Eur J Nucl Med Mol Imaging. (2025) 52:1489–99. doi: 10.1007/s00259-025-07306-y 40325259

[100] Clemente-Suárez VJ Li J Wang Y Zhang Y Liu X Zhao H . New insights and potential therapeutic interventions in metabolic diseases. Int J Mol Sci. (2023) 24:10892. doi: 10.3390/ijms241310892 37445852 PMC10342188

[101] Shitara H Li J Wang Y Zhang Y Liu X Zhao H . Relationship between muscle tone and elasticity: simultaneous quantitative assessment using train-of-four monitoring and continuous shear wave elastography during anesthesia induction—a prospective observational study. Diag (Basel). (2025) 15:689. doi: 10.3390/diagnostics15030293 39941223 PMC11817420

[102] Ma S Wang Y Zhang Y Li Y Liu X Zhao H . Dynamic characterization of single cells based on temporal cellular mechanical properties. IEEE Trans Nanobiosci. (2023) 22:19–27. doi: 10.1109/TNB.2021.3136198 34941515

[103] Xiao J Li J Wang Y Zhang Y Liu X Zhao H . Dynamic contrast-enhanced breast MRI features correlate with invasive breast cancer angiogenesis. NPJ Breast Cancer. (2021) 7:71. doi: 10.1038/s41523-021-00247-3 33863924 PMC8052427

[104] Liu Z Li J Wang Y Zhang Y Liu X Zhao H . Multi-stage mechanisms of tumor metastasis and therapeutic strategies. Signal Transduc Targeted Ther. (2024) 9:270. doi: 10.1038/s41392-024-01955-5 39389953 PMC11467208

[105] Zhang W Li J Wang Y Zhang Y Liu X Zhao H . Multi-feature enhancement fusion network for remote sensing image semantic segmentation. Sci Rep. (2026) 16:5023. doi: 10.1038/s41598-026-35723-y 41521236 PMC12877183

[106] Kreher R Li J Wang Y Zhang Y Liu X Zhao H . Multilabel segmentation and analysis of skeletal muscle and adipose tissue in routine abdominal CT scans. Comput Biol Med. (2025) 186:109622. doi: 10.1016/j.compbiomed.2024.109622 39778239

[107] Saito Y Li J Wang Y Zhang Y Liu X Zhao H . Classification of soybean chemical characteristics by excitation emission matrix coupled with t-SNE dimensionality reduction. Spectrochimica Acta Part A Mol Biomol Spectrosc. (2024) 322:124785. doi: 10.1016/j.saa.2024.124785 39008929

[108] Ryu J Li J Wang Y Zhang Y Liu X Zhao H . SegR-Net: a deep learning framework with multi-scale feature fusion for robust retinal vessel segmentation. Comput Biol Med. (2023) 163:107132. doi: 10.1016/j.compbiomed.2023.107132 37343468

[109] Wang S Li J Wang Y Zhang Y Liu X Zhao H . Automatic segmentation of lumbar spine MRI images based on improved attention U-Net. Comput Intell Neurosci. (2022) 2022:4259471. doi: 10.1155/2022/4259471 36156962 PMC9492365

[110] Wang Z Li J Wang Y Zhang Y Liu X Zhao H . Secure multi-party test case data generation through generative adversarial networks. Sci Rep. (2026) 16:5085. doi: 10.1038/s41598-026-35773-2 41526446 PMC12877002

[111] Kang L Li J Wang Y Zhang Y Liu X Zhao H . Tumor necrosis factor-α–dependent inflammation upregulates high mobility group box 1 to induce tumor promotion and anti–programmed cell death protein-1 immunotherapy resistance in lung adenocarcinoma. Lab Invest. (2025) 105:102164. doi: 10.1016/j.labinv.2024.102164 39461427

[112] Younis NN Li J Wang Y Zhang Y Liu X Zhao H . Inactivation of Wnt/β-catenin/renin angiotensin axis by tumor necrosis factor-alpha inhibitor, infliximab, ameliorates CKD induced in rats. Biochem Pharmacol. (2021) 185:114426. doi: 10.1016/j.bcp.2021.114426 33482150

[113] Foster MA Lunn MPT Carr AS . First-line immunosuppression in neuromuscular diseases. Pract Neurol. (2023) 23:327. doi: 10.1136/pn-2023-003708 37173131

[114] Aliazis K Scotti A Losa M Bertolaso L Delledonne M Anichini A . The tumor microenvironment’s role in the response to immune checkpoint blockade. Nat Cancer. (2025) 6:345–58. doi: 10.1038/s43018-025-00986-3 40514448 PMC12317369

[115] Yee AMF . Durable medication-free remission of sarcoidosis following discontinuation of anti-tumor necrosis factor-α therapy. Respir Med. (2023) 206:107055. doi: 10.1016/j.rmed.2022.107055 36459954

[116] Ma S Wang Y Zhang Y Li Y Liu X Zhao H . Quantitative characterization of cell physiological state based on dynamical cell mechanics for drug efficacy indication. J Pharm Anal. (2023) 13:388–402. doi: 10.1016/j.jpha.2023.02.004 37181289 PMC10173291

[117] Stadtmauer EA Fraietta JA Davis MM Cohen AD Weber KL Pantel D . CRISPR-engineered T cells in patients with refractory cancer. Science. (2020) 367:1455–60. doi: 10.1126/science.aba7365 32029687 PMC11249135

[118] Feng X Li J Wang Y Zhang Y Liu X Zhao H . CRISPR/Cas9 technology for advancements in cancer immunotherapy: from uncovering regulatory mechanisms to therapeutic applications. Exp Hematol Oncol. (2024) 13:102. doi: 10.1186/s40164-024-00570-y 39427211 PMC11490091

[119] Kawasaki Y Matsumoto K Suzuki R Kuroda J Hata H Ishii T . Practical insights into bispecific antibody therapy in multiple myeloma. Expert Rev Anticancer Ther. (2024) 24:1209–19. doi: 10.1080/14737140.2024.2445145 39729045

[120] Yang M Li J Wang Y Zhang Y Liu X Zhao H . Survival strategies: how tumor hypoxia microenvironment orchestrates angiogenesis. Biomed Pharmacotherapy. (2024) 176:116783. doi: 10.1016/j.biopha.2024.116783 38796970

[121] Slavin MB Khemraj P Hood DA . Exercise, mitochondrial dysfunction and inflammasomes in skeletal muscle. Biomed J. (2024) 47:100636. doi: 10.1016/j.bj.2023.100636 37499756 PMC10828562

[122] Li J Wang Y Zhang Y Liu X Zhao H Chen X . Draconis Sanguis (DS) from the fruit of Daemonorops draco Bl. ameliorates cardiac function through optimizing myocardial energy metabolism by promoting angiogenesis in ischemic heart failure. Phytomedicine. (2025) 140:156583. doi: 10.1016/j.phymed.2025.156583 40085987

[123] Zhai J Zhang Y Li J Liu X Zhao H Chen X . Chemotherapeutic and targeted drugs-induced immunogenic cell death in cancer models and antitumor therapy: an update review. Front Pharmacol. (2023) 14:1152934. doi: 10.3389/fphar.2023.1152934 37153795 PMC10160433

[124] Wu S Li J Wang Y Zhang Y Liu X Zhao H . Interleukin-6 (IL-6)-associated tumor microenvironment remodelling and cancer immunotherapy. Cytokine Growth Fac Rev. (2025) 85:93–102. doi: 10.1016/j.cytogfr.2025.01.001 39828476

[125] Islam F Rahman M Uddin M Hassan M Islam M Akter S . The association of cytokines IL-2, IL-6, TNF-α, IFN-γ, and IL-10 with the disease severity of COVID-19: a study from Bangladesh. Cureus. (2024) 16:e57610. doi: 10.7759/cureus.57610 38707035 PMC11069400

[126] Rahman T Islam F Karim M Uddin M Hassan M Akter S . Cytokines and their role as immunotherapeutics and vaccine adjuvants: the emerging concepts. Cytokine. (2023) 169:156268. doi: 10.1016/j.cyto.2023.156268 37320965

[127] Mirlekar B . Tumor promoting roles of IL-10, TGF-β, IL-4, and IL-35: its implications in cancer immunotherapy. SAGE Open Med. (2022) 10:20503121211069012. doi: 10.1177/20503121211069012 35096390 PMC8793114

[128] Sinha A Ghosh D Karati D . Tumor microenvironment and immunotherapy: from bench to bedside. Med Oncol. (2025) 42:244. doi: 10.1007/s12032-025-02818-x 40483667

[129] García-Pérez G Sánchez-Ramón N Martín-Pérez J López-Jiménez J García-Ruíz C Fernández-Checa JC . Generation and biological characterization of an anti-IL-6Rα biosimilar candidate antibody. Biotechnol J. (2025) 20:e70017. doi: 10.1002/biot.70017 40524396

[130] Blay JY Le Cesne A Penel N Boudou-Rouquette P Italiano A Toulmonde M . Anti-IL-6R Ab tocilizumab to treat paraneoplastic inflammatory syndrome of solid cancers. ESMO Open. (2025) 10:104088. doi: 10.1016/j.esmoop.2024.104088 39754984 PMC11758126

[131] Cheng A Holland SM . Anti-cytokine autoantibodies: mechanistic insights and disease associations. Nat Rev Immunol. (2024) 24:161–77. doi: 10.1038/s41577-023-00933-2 37726402

[132] Tomala J Cao SD Spangler JB . Engineering anticytokine antibodies for immune modulation. J Immunol. (2024) 212:225–34. doi: 10.4049/jimmunol.2300467 38166248

[133] Beecher G Kao A Levy JA Amato AA Greenberg HS Pittock SJ . Immune checkpoint inhibitor myopathy. Neurology. (2024) 103:e210031. doi: 10.1212/WNL.0000000000207654 39514829 PMC12309512

[134] Thompson JA Adamson J Anderson AR Baird K Basch E Chap L . NCCN guidelines® Insights: management of immunotherapy-related toxicities, version 2.2024. J Natl Compr Canc Netw. (2024) 22:582–92. doi: 10.6004/jnccn.2024.0057 39536465

[135] Ageeva T Rizvanov A Mukhamedshina Y . NF-κB and JAK/STAT signaling pathways as crucial regulators of neuroinflammation and astrocyte modulation in spinal cord injury. Cells. (2024) 13:1896. doi: 10.3390/cells13070581 38607020 PMC11011519

[136] Penna F Rubini G Costelli P . Immunomodulation: a new approach to cancer cachexia, potentially suitable for aging. Mol Aspects Med. (2024) 100:101318. doi: 10.1016/j.mam.2024.101318 39260232

[137] Calle-Ciborro B Sanchez-de-Toledo J Martin-Castro ME Garcia-Rodriguez A de la Fuente MA Bragado R . Pharmacological inhibition reveals participation of the endocytic compartment in positive feedback IL-6 secretion in human skeletal myotubes. Eur J Pharmacol. (2024) 984:177055. doi: 10.1016/j.ejphar.2024.177055 39395584

[138] Silva JG da Silva AS de Oliveira MC de Souza RE Santos AS Pereira LM . Resistance training and cardiovascular health: epigenetic regulation. Front Physiol. (2025) 16:1589241. doi: 10.3389/fphys.2025.1701689 41624015 PMC12851980

[139] Faiad J de Souza Lopes R de Oliveira Lima K da Silva Vaz I de Oliveira Carvalho K de Souza Santos R . Muscle loss in cancer cachexia: what is the basis for nutritional support? Front Pharmacol. (2025) 16:1519278. doi: 10.3389/fphar.2025.1519278 40078277 PMC11897308

[140] Chung JY Lee JH Park JS Kim YS Lee SH Park SJ . Sarcopenia: how to determine and manage. Knee Surg Relat Res. (2025) 37:12. doi: 10.1186/s43019-025-00265-6, PMID: 40098209 PMC11912661

[141] Menezes Junior ADS da Silva AS de Oliveira MC de Souza RE Santos AS Pereira LM . T-cell-driven immunopathology and fibrotic remodeling in hypertrophic cardiomyopathy: a translational scoping review. Cells. (2025) 15:218. doi: 10.3390/cells15010061 41511345 PMC12785778

[142] Cheon SY Lee JH Park JS Kim YS Lee SH Park SJ . The immune-inflammatory responses on the hypothalamic-pituitary-adrenal axis and the neurovascular unit in perioperative neurocognitive disorder. Exp Neurol. (2025) 386:115146. doi: 10.1016/j.expneurol.2025.115146 39805464

[143] Tran SD Smith A Jones B Brown C Davis D Evans E . Weight and blood-based markers of cachexia predict disability, hospitalization and worse survival in cancer immunotherapy patients. J Cachexia Sarcopenia Muscle. (2025) 16:e13685. doi: 10.1002/jcsm.13685 39817619 PMC11736629

[144] Perner F Muller A Schmidt C Weber M Hoffmann R Brandts C . Malignant JAK-signaling: at the interface of inflammation and Malignant transformation. Leukemia. (2025) 39:1011–30. doi: 10.1038/s41375-025-02569-8 40140631 PMC12055591

[145] Lu Y Zhang Y Wang Y Li J Liu X Zhao H . Immunometabolism of Tregs: mechanisms, adaptability, and therapeutic implications in diseases. Front Immunol. (2025) 16:1536020. doi: 10.3389/fimmu.2025.1536020 39917294 PMC11798928

[146] Li Y Zhang Y Wang Y Li J Liu X Zhao H . Mitigating tumor heterogeneity in lung cancer: a dual-targeted NIR-II imaging strategy for accurate tumor localization and intraoperative navigation. Cancer Lett. (2026) 640:218241. doi: 10.1016/j.canlet.2026.218241 41490754

[147] Onishi K Tanaka Y Suzuki T Sato K Kobayashi M Yamamoto Y . Environmental enrichment enhances anesthetic actions in rat amygdala hippocampal circuits *in vitro*. Front Pharmacol. (2025) 16:1732630. doi: 10.3389/fphar.2025.1732630 41487527 PMC12757428

